# A Survey of Data Quality Measurement and Monitoring Tools

**DOI:** 10.3389/fdata.2022.850611

**Published:** 2022-03-31

**Authors:** Lisa Ehrlinger, Wolfram Wöß

**Affiliations:** ^1^Institute for Application-Oriented Knowledge Processing (FAW), Johannes Kepler University, Linz, Austria; ^2^Software Competence Center Hagenberg GmbH, Hagenberg, Austria

**Keywords:** data quality, data quality tools, data quality measurement, data quality monitoring, data profiling, information quality

## Abstract

High-quality data is key to interpretable and trustworthy data analytics and the basis for meaningful data-driven decisions. In practical scenarios, data quality is typically associated with data preprocessing, profiling, and cleansing for subsequent tasks like data integration or data analytics. However, from a scientific perspective, a lot of research has been published about the measurement (i.e., the detection) of data quality issues and different generally applicable data quality dimensions and metrics have been discussed. In this work, we close the gap between data quality research and practical implementations with a detailed investigation on *how data quality measurement and monitoring concepts are implemented in state-of-the-art tools*. For the first time and in contrast to all existing data quality tool surveys, we conducted a systematic search, in which we identified 667 software tools dedicated to “data quality.” To evaluate the tools, we compiled a requirements catalog with three functionality areas: (1) data profiling, (2) data quality measurement in terms of metrics, and (3) automated data quality monitoring. Using a set of predefined exclusion criteria, we selected 13 tools (8 commercial and 5 open-source tools) that provide the investigated features and are not limited to a specific domain for detailed investigation. On the one hand, this survey allows a critical discussion of concepts that are widely accepted in research, but hardly implemented in any tool observed, for example, generally applicable data quality metrics. On the other hand, it reveals potential for functional enhancement of data quality tools and supports practitioners in the selection of appropriate tools for a given use case.

## 1. Introduction

Data quality (DQ) measurement is a fundamental building block for estimating the relevance of data-driven decisions. Such decisions accompany our everyday life, for instance, machine-based decisions in ranking algorithms, industrial robots, and self-driving cars in the emerging field of artificial intelligence. The negative impact of poor data on the error rate of machine learning (ML) models has been shown by Sessions and Valtorta (2006) and Ehrlinger et al. (2019). Also human-based decisions rely on high-quality data, for example, the decision whether to promote or to suspend the production of a specific product is usually based on sales data. Despite the clear correlation between data and decision quality, 84 % of the CEOs in the US are concerned about their DQ (KPMG International, 2016) and “organizations believe poor data quality to be responsible for an average of $15 million per year in losses” (Moore, 2018). Thus, DQ is “no longer a question of ‘hygiene' [...], but rather has become critical for operational excellence” and is perceived as the greatest challenge in corporate data management (Otto and Österle, 2016).

To increase the trust in data-driven decisions, it is necessary to measure, know, and improve the quality of the employed data with appropriate tools (Ehrlinger et al., 2018; Heinrich et al., 2018). DQ improvement (i.e., data cleansing), which is based on DQ measurement, are both part of comprehensive DQ management. Most existing methodologies describe DQ management as cyclic process, which is carried out continuously (cf. Redman, 1997; Wang, 1998; English, 1999; Lee et al., 2009; Sebastian-Coleman, 2013). Yet, according to a German survey, 66 % of companies use Excel or Access solutions to validate their DQ and 63 % of the companies determine their DQ manually and *ad hoc* without any long-term DQ management strategy (Schäffer and Beckmann, 2014). Considering such studies and the increasing amount of data to be processed, there is a clear need for intensive research to automate DQ management tasks. Sebastian-Coleman (2013) also states that “without *automation*, the speed and volume of data will quickly overwhelm even the most dedicated efforts to measure.”

Research about data quality has been conducted since the 1980s and since then, DQ is most often associated with the “fitness for use” principle (Chrisman, 1983; Wang and Strong, 1996), which refers to the subjectivity and context-dependency of this topic. Data quality is typically referred to as a multi-dimensional concept, where single aspects are described by DQ *dimensions* (e.g., accuracy, completeness, timeliness). The fulfillment of a DQ dimension can be quantified using one or several DQ *metrics* (Ehrlinger et al., 2018). According to the IEEE standard (IEEE, 1998), a metric is a formula that yields a numerical value. In parallel to the scientific background, a wide variety of commercial, open-source, and academic DQ tools with different foci have been developed since then, in order to support the automation of DQ management. The range of functions offered by those tools varies widely, because the term “data quality” is context-dependent and not always used consistently. Despite the large number of publications, tools, and concepts into data quality, it is not always clear how to map the concepts from the theory (i.e., dimensions and metrics) to a practical implementation (i.e., tools). Therefore, the question of how to measure and monitor DQ automatically is still not sufficiently answered (Sebastian-Coleman, 2013). In this survey, we contribute to this research question by providing a detailed investigation of DQ measurement and monitoring functionalities in state-of-the-art DQ tools.

Specifically, we conducted a systematic search, where we identified 667 software tools dedicated to “data quality.” According to predefined exclusion criteria, we selected 13 DQ tools (8 commercial and 5 open-source tools) for deeper investigation. To systematically evaluate the functional scope of the tools, we introduce a requirements catalog comprising three categories: (1) data profiling, (2) DQ measurement in terms of dimensions and metrics, and (3) continuous DQ monitoring. Since the focus of this survey is on the automation of DQ tasks, we specifically observe the measurement capabilities (i.e., how to detect and report DQ issues) and to which extent the tools support automated DQ monitoring, required to ensure high-quality data over time (Ehrlinger and Wöß, 2017). We deliberately exclude tools that solely offer data cleansing and improvement functions, because an automated modification of the data (i.e., data cleansing) is usually not possible in productive information systems with critical content. Consequently, our main contributions can be summarizes as follows:

- To the best of our knowledge, we conducted the first *systematic search* to identify DQ tools and thus, give a comprehensive overview on the market.
- We compiled a *requirements catalog* to investigate data profiling, DQ measurement, and automated DQ monitoring functionalities of DQ tools. This catalog summarizes and classifies tasks that are required for automated and continuous DQ measurement in a new way and supports follow-up studies, e.g., on domain-specific DQ tools.
- Based on the *detailed investigation* of 13 DQ tools, we propose a new research direction for DQ measurement and highlight potential for enhancement in the DQ tools.

The results of this survey are not only relevant for DQ professionals to select the most appropriate tool for a given use case, but also highlight the current capabilities of state-of-the-art DQ tools. Especially since such a wide variety of DQ tools exist, it is often not clear which functional scope can be expected. The main findings of this article can be summarized as follows:

- Despite the presumption that the emerging market of DQ tools is still under development (cf. Selvage et al., 2017), we found a vast number (667) of DQ tools through our systematic search, where most of them have never been included in one of the existing surveys.
- Approximately half (50.82 %) of the DQ tools were domain specific, which means they were either dedicated to specific types of data or built to measure the DQ of a proprietary tool.
- 16.67 % of the DQ tools focused on data cleansing without a proper DQ measurement strategy (i.e., measurements are used to modify the data, but no comprehensive reports are provided).
- Most surveyed tools supported data profiling to some extent, but considering the research state, there is potential for functional enhancement in data profiling, especially with respect to multi-column profiling and dependency discovery.
- We did not find a tool that implements a wider range of DQ metrics for the most important DQ dimensions as proposed in research papers (cf. Piro, 2014; Batini and Scannapieco, 2016; Heinrich et al., 2018). Identified metric implementations have several drawbacks: some are only applicable on attribute-level (e.g., no aggregation), some require a gold standard that might not exist, and some have implementation errors.
- In general-purpose DQ tools, DQ monitoring is considered a premium feature, which is liable to costs and only provided in professional versions. Exceptions are dedicated open-source DQ monitoring tools, like Apache Griffin or MobyDQ, which support the automation of rules, but lack predefined functions and data profiling capabilities.

This article is structured as follows: Section 2 summarizes related research concerning DQ management, measurement, and monitoring. Section 3 covers the applied methodology to conduct this research, including related surveys, our research questions, and the tool selection strategy. Based on the existing research from Section 2, we introduce a new requirements catalog to evaluate DQ tools and the accompanying evaluation strategy in Section 4. In Section 5, we describe the tools, which have been selected for investigation, and discuss the evaluation. The results and lessons learned are summarized in Section 6. We conclude in Section 7 with an outlook on future work.

## 2. Theoretical Background on Data Quality

Despite different existing interpretations, the term “data quality” is most frequently described as “fitness for use” (Chrisman, 1983; Wang and Strong, 1996), referring to the high subjectivity and context-dependency of this topic. Information quality is often used as synonym for data quality and even though both terms can be clearly distinguished, because “data” refers to plain facts and “information” describes the extension of those facts with context and semantics, they are often used interchangeably in the DQ literature (Wang, 1998; Zhu et al., 2014). We use the term data quality because our focus is on processing objectively, automatically retrievable facts (i.e., intrinsic data characteristics). The term information serves as synonym for data in the systematic search to achieve higher coverage.

### 2.1. Data Quality Management

The Data Management Association (DAMA) defines “data quality management” as the analysis, improvement and assurance of data quality (Otto and Österle, 2016). Over the years, a number of different DQ methodologies (also declared as “frameworks,” “programs,” or “methods”) have been proposed, for example, TDQM (Total Data Quality Management) by Wang (1998), AIMQ (A Methodology for Information Quality Assessment) by Lee et al. (2002), and the DQ assessment methods by Pipino et al. (2002) and Maydanchik (2007). Batini et al. (2009) conducted a comprehensive comparison of DQ methodologies in 2009, and Cichy and Rass (2019) provide a recent overview on generally applicable DQ methodologies in 2019. Although these methodologies have different characteristics and emphases, it is possible to extract four core activities (cf. English, 1999; Maydanchik, 2007; Batini et al., 2009; Cichy and Rass, 2019): (1) state reconstruction, (2) DQ measurement or assessment, (3) data cleansing or improvement, and (4) the establishment of continuous DQ monitoring. Not all methodologies include all of these steps, for example, step (1) is omitted by Maydanchik (2007) and step (4) is omitted in the DQ methodology survey by Batini et al. (2009). Further, some methodologies include additional activities like monitoring of data integration interfaces (cf. Maydanchik, 2007), which we do not consider because of their specialization. In the following paragraphs, we describe the four core steps of a DQ methodology in detail to clarify the difference between DQ measurement, DQ monitoring, and data cleansing activities, where the latter ones are not included in the survey. Step (1), the state reconstruction, describes the collection of contextual information on the observed data, as well as on the organization where a DQ project is carried out (Batini et al., 2009). Since the focus of this article is on DQ tool functionalities, we restrict step (1) in the following to the data part (i.e., data profiling) and do not describe gathering of contextual information on the organization in detail.

#### 2.1.1. Data Profiling

Data profiling is described as the process of analyzing a dataset to collect data about data (i.e., metadata) using a broad range of techniques (Naumann, 2014; Abedjan et al., 2015, 2019). Thus, it is an essential task prior to any DQ measurement or monitoring activity to get insight into a given dataset. Exemplary information that is gathered during data profiling are the number of distinct or missing (i.e., null) values in a column, data types of attributes, or occurring patterns and their frequency (e.g., formatting of telephone numbers) (Abedjan et al., 2015). We refer to Abedjan et al. (2015, 2019) for a detailed discussion on data profiling techniques and tasks. According to Selvage et al. (2017) and the findings of our survey, most general-purpose DQ tools offer data profiling capabilities to some extent.

#### 2.1.2. Data Quality Measurement

According to Sebastian-Coleman (2013), one of the biggest challenges for DQ practitioners is to answer the question on how data quality should be actually measured. Ge and Helfert (2007) indicate that this is also true for the synonymously used term *assessment* by stating that one of the major questions in DQ research is “How to assess data quality?.” The term *measure* describes “to ascertain the size, amount, or degree of something by using an instrument or device marked in standard units or by comparing it with an object of known size” (McKean, 2005). Although the term *assessment* is often used as synonym for measurement, especially in DQ literature there is a clear distinction between both terms. Assessment is the “evaluation or estimation of the nature, ability, or quality of something” and extends the concept of measurement by evaluating the measurement results and drawing a conclusion about the object of assessment (McKean, 2005; Sebastian-Coleman, 2013). DQ assessment is also described as the detection and initial estimation of data quality as well as the impact analysis of occurring DQ problems (English, 1999; Apel et al., 2015). In this survey, we use the term *measurement* since the focus is on measurement capabilities of DQ tools, independently of the interpretation of the results by a user.

In addition to scientific publications, standards should represent the consensus of practitioners and researchers likewise. In terms of data quality, there has been considerable work done by the ISO/IEC JTC 1 (“Information technology,”) subcommittee 7 on “software and systems and engineering.” SC 7's working group 06 published (ISO/IEC 25012:2008, 2008; ISO/IEC 25040:2011, 2011; ISO/IEC 25024:2015(E), 2015). In parallel, subcommittee SC 4 “Industrial data” of the technical committee ISO/TC 184 (“Industrial automation systems and integration”) published (ISO 8000-8:2015(E), 2015). While (ISO 8000-8:2015(E), 2015) defines prerequisites for the measurement and reporting of information and data quality on a very general level, (ISO/IEC 25012:2008, 2008) provides more concrete DQ measures as well as an explanation of how to apply them. According to ISO 8000-8:2015(E) (2015), data can be measured on a very general level according to (1) *syntactic quality* that describes the degree to which data conforms to a specified syntax, (2) *semantic quality*, ie., the degree to which data corresponds to its real representation, or (3) *pragmatic quality*, i.e., the degree to which data is suitable for a specific purpose. ISO/IEC 25012:2008 (2008) defines the “measurement” (of data quality) as “set of operations having the object of determining a value of a measure” and define a set of normalized quality measures (between 0 and 1).

The partition of data quality into a set of DQ dimensions, which can be measured with metrics, is widely accepted in DQ research (cf. Wang and Strong, 1996; Lee et al., 2009; Batini and Scannapieco, 2016). For example, Lee et al. (2009) state that “DQ assessment requires assessments along a number of dimensions.” The quality measures provided by ISO/IEC 25012:2008 (2008) correspond to the most popular metrics in literature (e.g., accuracy, completeness, consistency). Despite the wide agreement on DQ dimensions and metrics (i.e., measures) in general and a lot of research over the last decades, there is still no consensus on a standardized list of dimensions and metrics for DQ measurement (Sebastian-Coleman, 2013; Myers, 2017). Thus, we observe existing DQ dimensions and metrics and justify their inclusion in our requirements catalog in Section 2.2.

#### 2.1.3. Data Cleansing

Data cleansing describes process of correcting erroneous data or data glitches (Dasu and Johnson, 2003). In practice, automatable cleansing tasks include customer data standardization, de-duplication, and matching. Other efforts to improve DQ are usually performed manually. While automated data cleansing methods are very valuable for large amounts of data, they pose risks to insert new errors that are rarely well understood (Maydanchik, 2007). We intentionally did not observe data cleansing functionalities in this survey, since the focus is on the detection of DQ problems. However, data cleansing algorithms are usually based on DQ measurement, since it is initially necessary to detect DQ problems to increase the quality of a given dataset.

#### 2.1.4. Data Quality Monitoring

The term “DQ monitoring” is mainly used implicitly in literature without an established definition and common understanding. This leads to different interpretations when the term is mentioned in scientific publications or by companies promoting and describing their DQ tool. There is a difference between “data monitoring,” which describes continuous checking of rules, and “DQ monitoring,” which is ongoing measurement of DQ (Ehrlinger and Wöß, 2017). The aim of this survey is to observe not only the functionalities of current DQ tools in terms of data profiling and measurement, but also in terms of true DQ monitoring. Pushkarev et al. (2010) and a follow-up study (Pulla et al., 2016) point out that none of the tools observed had any monitoring functionality. We however want to include this criterion in our requirements catalog since there is evidence on several DQ tool websites that they do offer monitoring functionalities, but have not been observed by Pushkarev et al. (2010) and Pulla et al. (2016). According to the ISO standard 8,000 (ISO 8000-8:2015(E), 2015), pragmatic data quality measurement requires interaction with the respective users who validate the data. Consequently, fully automated DQ monitoring is restricted to syntactic and semantic DQ aspects.

### 2.2. Data Quality Dimensions and Metrics

Data quality is often described as concept with multiple dimensions, so that every DQ dimension refers to a specific aspect of the quality of data (Ehrlinger and Wöß, 2019). Over the years, a wide variety of dimensions and dimension classifications have been proposed (Ballou and Pazer, 1985; Wand and Wang, 1996; Wang and Strong, 1996; Pipino et al., 2002; Ge and Helfert, 2007; Batini and Scannapieco, 2016). An overview of possible dimensions and classifications is provided by Laranjeiro et al. (2015); Scannapieco and Catarci (2002). Despite intensive research and an ongoing discussion on DQ dimensions, there is still no consensus on which dimensions are the essence for DQ measurement (Sebastian-Coleman, 2013). Our evaluation framework covers the four most frequently used dimensions accuracy, completeness, consistency, and timeliness (Wand and Wang, 1996; Scannapieco and Catarci, 2002; Hildebrand et al., 2015).

Piro (2014) distinguishes between “hard dimensions” (including accuracy, completeness, and timeliness, amongst others), which can be measured objectively using check routines, and “soft dimensions,” which can only be assessed using subjective evaluation. However, also objective check routines require a preceding subjective and domain-specific definition of the data objects to be measured, in order to consequently follow the “fitness for use” approach (Piro, 2014).

In conjunction with the discussion of DQ dimensions, it is often mentioned that the definition of specific DQ metrics is required to apply those dimensions in practice. A metric is a function that maps a quality dimension to a numerical value, which allows an interpretation of a dimension's fulfillment (IEEE, 1998). Such a DQ metric can be measured on different aggregation levels: on value-level, column or attribute-level, tuple or record-level, table or relation-level, as well as database (DB)-level (Hildebrand et al., 2015). The aggregation could, for example, be performed with the weighted arithmetic mean of the calculated metric results from the previous level (e.g., results of the record-level to calculate the table-level metric) (Hinrichs, 2002). Heinrich et al. (2018) proposed five requirements for DQ metrics to ensure reliable decision-making: “the existence of minimum and maximum metric values (R1), the interval scaling of the metric values (R2), the quality of the configuration parameters and the determination of the metric values (R3), the sound aggregation of the metric values (R4), and the economic efficiency of the metric (R5).” However, other researchers claim “that a more general approach is required” (Bronselaer et al., 2018) to assess the usefulness and validity of a DQ metric. In the following, we describe four prominent DQ dimensions along with common metrics for their calculation. The list of metrics is not exhaustive, but should give an impression about the research conducted in this area since we observe the existence of such or similar metrics in our DQ tool evaluation.

#### 2.2.1. Accuracy

Although accuracy is sometimes described as the most important data quality dimension, a number of different definitions exist (Wand and Wang, 1996; Haegemans et al., 2016). In DQ literature, accuracy can be described as the closeness between an information system and the part of the real-world it is supposed to model (Batini and Scannapieco, 2016). From the natural sciences perspective, accuracy is usually defined as the “magnitude of an error” (Haegemans et al., 2016). We refer to Haegemans et al. (2016) for a detailed discussion on the definitions of accuracy and a comprehensive list of metrics related to accuracy. Here, we provide a few exemplary metrics. Redman (2005) defines field- and record-level accuracy as follows:


(1) $$\begin{array}{l} \text{field level accuracy }=\frac{\text{number of fields judged }"\text{correct}"}{\text{number of fields tested}} \end{array}$$

(Redman, 2005),


(2) $$\text{record level accuracy }=\frac{\text{number of records judged }"\text{completely correct}"}{\text{number of records tested}}$$

This metric is also reused by the DAMA UK (Askham et al., 2013) by generalizing “fields” and “records” to “objects.” Lee et al. (2009) use the inverse metric ($1-\frac{\text{Number of data units in error}}{\text{Total number of data units}}$) and Fisher et al. (2009) additionally take into account the randomness of the occurrence of an error *ROE* and the probability distribution of the occurrence of an error *PDOE*:


(3) $$\begin{array}{l} \text{accuracy}=\left(\frac{\text{NrOfCorrectValues}}{\text{TotalNrOfValues}},\text{ROE},\text{PDOE}\right) \end{array}$$

(Fisher et al., 2009).

Hinrichs (2002) proposed the accuracy metric in Equation (4), which can be aggregated on different levels. On attribute-value-level, the metric *Q*_*Gen*_ for accuracy (*Gen* is “Genauigkeit” in German, which means “accuracy” in English) is defined by the ratio between a value's arity and its optimal arity for numeric values. For a numeric attribute *A*, *s*_*opt*_(*A*) is the optimal number of digits and decimals for *A*, *w* is a value of *A* and *s*(*w*) is the actual number of digits and decimals for *w* in attribute *A*. Since *s*_*opt*_(*A*) is not necessarily maximal, the metric needs to be normalized by [0, 1] (Hinrichs, 2002).


(4) $$\begin{array}{l} Q_{Gen}\left(w,A\right)=min\left(\frac{s\left(w\right)}{s_{opt}\left(A\right)},1\right) \end{array}$$

Hinrichs, (2002).

For non-numeric attributes, Hinrichs (2002) suggests to assign *w* to plane *i* within a classification *K* with *n* planes (*K*_1_, ..., *K*_*n*_) and to replace *s*(*w*) with *i* and to select *s*_*opt*_(*A*) from *K* with *s*_*opt*_(*A*) ≤ *n*. For a tuple *t*, accuracy *Q*_*Gen*_ is measured according to:


(5) $$\begin{array}{l} Q_{Gen}\left(t\right)=\frac{\sum_{j=1}^{n}Q_{Gen}\left(t.A_{j},A_{j}\right)g_{j}}{\sum_{j=1}^{n}g_{j}} \end{array}$$

Hinrichs, (2002),

where *t*.*A*_1_, ..., *t*.*A*_*n*_ are the attribute values for attributes *A*_1_, ..., *A*_*n*_ that specify the observed tuple *t*. Factor *g*_*j*_ is the relative importance of *A*_*j*_ with respect to the total tuple and is an expert-defined weight (Hinrichs, 2002). The accuracy on table-level is then calculated as the arithmetic mean of the tuple accuracy measurements, and the accuracy on DB-level is the arithmetic mean of the table-level accuracy measurements. For a more detailed discussion on the metric, we refer to Hinrichs (2002).

#### 2.2.2. Completeness

Completeness is very generally described as the “breadth, depth, and scope of information contained in the data” (Wang and Strong, 1996; Batini and Scannapieco, 2016) and covers the condition for data to exist. Considering related work (cf. Redman, 1997; Hinrichs, 2002; Lee et al., 2009; Ehrlinger et al., 2018), the most generic metric for completeness can be defined as:


(6) $$\begin{array}{l} \text{Completeness}=\frac{\left|e_{c}\right|}{\left|e\right|}, \end{array}$$

where |*e*_*c*_| is the number of complete elements and |*e*| is the total number of elements. Here, the generic term “element” can refer to any data unit, e.g., an attribute, a record, or a table. Lee et al. (2009) use the inverse metric ($1-\frac{\text{Number of incomplete elements}}{\text{Total number of elements}}$ Lee et al., 2009) and Batini and Scannapieco (2016) suggest comparing the number of complete elements to the (total) number of elements in a perfect reference dataset. A more detailed specification on how to calculate completeness is provided by Hinrichs (2002), who assigns 0.0 to a field value that is null or equivalent and 1.0 else. Based on this assumption, completeness can be calculated analogously to the accuracy metric on different aggregation levels with the weighted arithmetic mean. For example, the completeness *Q*_*Voll*_ (*Voll* is “Vollständigkeit” in German, which means “completeness” in English) on table-level is defined as:


(7) $$\begin{array}{l} Q_{Voll}\left(T\right)=\frac{\sum_{i=1}^{\left|T\right|}Q_{Voll}\left(t_{i}\right)}{\left|T\right|} \end{array}$$

Hinrichs, (2002),

where |*T*| is the number of records in table *T* and *Q*_*Voll*_(*t*_*i*_) is the completeness of record *t*_*i*_. We want to point out that in addition to the assumption by Hinrichs, who counts true missing values (i.e., null), it is also possible to approach completeness in a more rigorous way by considering default values or textual entries stating “NaN” (i.e., not a number) as incomplete values.

Although Hinrichs does not propose a completeness metric per attribute (i.e., column) and other related work like (Askham et al., 2013) describe attribute-level completeness only textually, such a metric can be derived from the description and Equation (6) as follows:


(8) $$\begin{array}{l} C_{att}=\frac{\left|v_{c}\right|}{\left|v\right|}, \end{array}$$

where |*v*| is the total number of values within a column and |*v*_*c*_| is the number of complete values that are not null.

#### 2.2.3. Consistency

There are also different definitions for the consistency dimension. According to Batini and Scannapieco (2016), “consistency captures the violation of semantic rules defined over data items, where items can be tuples of relational tables or records in a file.” An example for such rules are integrity constraints from the relational theory. Hinrichs (2002) assumes for his proposed consistency metric that domain knowledge is encoded into rules and excludes contradictions within the rules and fuzzy or probabilistic assumptions. Consequently the consistency *Q*_*Kon*_ (*Kon* is “Konsistenz” in German, which means “consistency” in English) of an attribute value *w* is defined as


(9) $$\begin{array}{l} Q_{Kon}(w)=\frac{1}{\sum_{j=1}^{n}r_{j}(w)g_{j}+1} \end{array}$$

Hinrichs, (2002),

where *g*_*j*_ is the degree of severity of *r*_*j*_(*w*), and *r*_*j*_(*w*) is the violation of consistency rule *r*_*j*_ (within a set of *n* consistency rules), applied to the attribute value *w*, and defined as


(10) $$\begin{array}{l} r_{j}(w)\{\begin{array}{ll} 0 & \text{if }w\text{ satisfies }r_{j}\\ 1 & \text{otherwise}. \end{array} \end{array}$$

Hinrichs, (2002)

Consistency rules cannot only be defined on attribute-value-level, but also on tuple-level. The calculation of the consistency on table- or database-level is in alignment to the accuracy and completeness metric calculated as the arithmetic mean of the tuple-level consistency (Hinrichs, 2002).

Sebastian-Coleman (2013) suggests measuring consistency over time by comparing the “record count distribution of values (column profile) to past instances of data populating the same field.”

#### 2.2.4. Timeliness

Timeliness describes “how current the data are for the task at hand” (Batini and Scannapieco, 2016) and is closely connected to the notions of *currency* (update frequency of data) and *volatility* (how fast data becomes irrelevant). A different definition states that “timeliness can be interpreted as the probability that an attribute value is still up-to-date” (Heinrich et al., 2007). A list of different metrics to calculate timeliness is provided by Heinrich and Klier (2009), where the authors suggest calculating timeliness based on the definition by Heinrich et al. (2007) according to:


(11) $$\begin{array}{l} Q_{Time.}^{\omega }\left(t\right):=\text{exp}\left(-\text{decline}\left(A\right)\cdot t\right) \end{array}$$

(Heinrich et al., 2007),

where ω is the considered attribute value and *decline*(*A*) is the decline rate, which specifies the average number of attributes that become outdated within the time period *t* (Heinrich et al., 2007).

This list of metrics for the DQ dimensions accuracy, completeness, timeliness, and consistency, is by no means exhaustive, but a comprehensive discussion would be out of scope for this article. We conclude that literature offers a number of specifically formulated metrics to measure DQ dimensions and this survey observes their implementation in state-of-the-art DQ tools.

### 2.3. Requirements for Data Quality Tools

In general, there are very few scientific papers that study the functional scope of DQ tools and even less papers that propose a dedicated requirements catalog for their evaluation. The differentiation of our DQ tool survey to existing ones (and consequently their requirements) is explained in detail in Section 3.1. In summary, the proposed requirements were of too less detail or with a different functional focus.

In addition to existing surveys, Goasdoué et al. (2007) explicitly proposed an evaluation framework for DQ tools without publishing the results of their evaluation. The proposed requirements were adapted to the context of the company, they performed the DQ tool evaluation for: Électricité de France (EDF), a French electric utility company, and more precisely to their CRM (customer relationship management) environments. Thus, the main differences to our requirements catalog are a more detailed evaluation of address normalization, duplicate detection, and reporting capabilities, but less details in data profiling and no coverage of DQ monitoring functionality.

In addition to requirements defined by researchers, there are several practitioner- and vendor-focused surveys by Gartner Inc. (cf. Judah et al., 2016; Selvage et al., 2017; Chien and Jain, 2019), which observe DQ tools by means of the following DQ capabilities: connectivity, data profiling, measurement and visualization, monitoring, parsing, standardization and cleaning, matching, linking and merging, multi-domain support, address validation/geocoding, data curation and enrichment, issue resolution and workflow, metadata management, DevOps environment, deployment environment, architecture and integration, and usability. Similarly, Loshin (2010) defines the following eight requirements a DQ tool must offer: “data profiling, parsing, standardization, identity resolution, record linkage and merging, data cleansing, data enhancement, and data inspection and monitoring.” Such lists of requirements were too coarse grained for our aim to specifically observe data profiling functionality, DQ measurement, and DQ monitoring functionality. While general features like connectivity and usability of the tools are not necessary to answer our research questions, we added a short textual description to each tool we observed.

## 3. Survey Methodology

A systematic survey is usually started by defining a “protocol that specifies the research questions being addressed and the methods that will be used” (Kitchenham, 2004). This section describes the protocol we developed to systematically conduct our survey. The structure of the protocol has been derived from the methodology for systematic reviews in computer science by Kitchenham (2004). Since the focus in Kitchenham (2004) is on the evaluation of primary research papers and not on specific implementations, we omit steps 5, 6, and 7 of the suggested planning information, including quality assessment, a data extraction strategy, and the synthesis of the extracted data from the original research papers.

### 3.1. Related Surveys

Although a lot of DQ methods and tools have been published, there are few scientific studies about the functional scope of DQ tools. Gartner Inc. (cf. Judah et al., 2016; Selvage et al., 2017; Chien and Jain, 2019) lists the strengths and cautions of vendors of commercial DQ tools in their “Magic Quadrant for Data Quality Tools” 2016 (17 vendors), 2017 (16 vendors), and 2019 (15 vendors). They include vendors that offer software tools or cloud-based services, which deliver general-purpose DQ functionalities, including at least profiling, parsing, standardization/cleansing, matching, and monitoring (Selvage et al., 2017). The study is vendor-focused and does not provide a detailed comparison of the respective data quality tools in terms of functionality (e.g., measurement and monitoring capabilities). However, the “Magic Quadrant for Data Quality Tools” contains a representative selection of commercial DQ tools, which is a valuable complement to our survey. The closest survey to our work in terms of tool comparison structure has been published by Fraunhofer IAO in German language (Kokemüller and Haupt, 2012). While (Kokemüller and Haupt, 2012) focus on tools popular in German, we at a scientific approach to observe the availability of DQ tools from a general perspective by also justifying the tool selection.

Woodall et al. (2014) categorize different methods to assess and improve DQ. They understand DQ methods as automatically executable algorithms to detect or correct a DQ problem, e.g., column analysis, data verification, or data standardization. As basis for their classification, they reviewed the list of DQ tools included in the “Magic Quadrant for Data Quality Tools 2012” by Gartner and extracted a list of DQ methods that tackle specific DQ problems. Woodall et al. (2014) do not provide an in-depth comparison of which method is contained in which tool since their focus is on the method classification.

Barateiro and Galhardas (2005) compared 9 academic and 28 commercial DQ tools in a scientific survey. This article does not cover state-of-the-art tools and the survey was not conducted in a systematic way, which means, it is unclear how the list of DQ tools has been selected. In addition, the authors state that DQ tools aim at detecting and correcting data problems, which is why they observe functionalities for both, DQ measurement as well as data cleansing, with an emphasis on the second aspect. In contrast, we focus on the measurement of data quality issues only, with special consideration of long-term monitoring functionality.

Pushkarev et al. (2010) proposed an overview of 7 open-source or freely available DQ tools. They described each tool briefly and compared the functionalities of the tools by means of *performance criteria* (including 6 usability features like data source connectivity, report creation, or the graphical user interface—GUI) and *core functionality*. The core functionality consists of 4 groups, which are further subdivided into specific features that are observed: data profiling (e.g., data pattern discovery), data integration (e.g., ETL), data cleansing (e.g., parsing and standardization), and data monitoring. Due to the limited number of pages, Pushkarev et al. do not provide detailed insights in the implementation of specific criteria, and mainly distinguish between the availability of a feature (Y) or its absence (N). For example, the authors list 9 usability criteria for the GUI, but in the evaluation they only distinguish between (g) representing “not user friendly GUI” and a (G) for “user-friendly GUI” with drag and drop functionality. Pulla et al. (2016) published a revised version of the tool overview, which is very similar to the original work in terms of structure and methodology. They used the same criteria structure as Pushkarev et al. (2010), but omitted the data monitoring group [since it is not provided by any of the tools according to Pulla et al. (2016)] and 4 other sub-features without further justification. The list of investigated DQ tools was extended from 7 to 10. Our survey differs notably from these two papers since, we conducted a systematic search to select DQ tools and also investigated commercial tools, while Pushkarev et al., 2010 and (Pulla et al., 2016) presented a predefined selection of free or open-source tools without publishing their selection strategy. Moreover, we focus on data profiling, DQ measurement, and DQ monitoring and evaluate these feature groups with a more detailed and comprehensive criteria catalog as provided by other published surveys mentioned above.

Another study by Gao et al. (2016) focuses on big data quality assurance. However, the authors did not clarify the methodology, that is, the selection of the investigated tools and evaluation criteria. In contrast to our survey, were the focus is on the actual DQ measurement functionalities, the comparison in Gao et al. (2016) includes mainly technical features like the supported operating system and data sources, as well as a limited list of 4 basic data validation functions.

Table 1 provides an overview on related DQ tool surveys and compares them to our work. It can be seen that there exists no other survey, which (1) conducted a systematic search to select the DQ tools for investigation, (2) addresses both practitioners and researchers, and (3) investigates data profiling, DQ measurement, DQ monitoring, as well as the vendors in terms of customer support. In contrast to other surveys that focus mainly on commercial or open-source tools, we provide a good digest of the market by investigating a total number of 13 DQ tools, from which five are open-source and eight commercial.

**Table 1:** Comparison of related data quality tool surveys.

				**No. of DQ Tools**			**Scope of Investigation**					
**Survey Authors and Year**	**Target Group**	**Survey Focus**	**Selection Strategy**	**Total number of tools**	**Open-source tools**	**Commercial tools**	**DQ tool vendors**	**Data profiling**	**DQ measurement**	**DQ monitoring**	**Data cleansing**	**Technical features**
Gartner Inc. – (Judah et al., 2016)	Practitioners	DQ tool vendors	By the authors	46	(1 / 45)		x					
Gartner Inc. – (Selvage et al., 2017)	Practitioners	DQ tool vendors	By the authors	39	(1 / 38)		x					
Gartner Inc. – (Chien and Jain, 2019)	Practitioners	DQ tool vendors	By the authors	26	(1 / 25)		x					
Kokemüller and Haupt (2012)	Practitioners	German DQ tools	By the authors	17	(0 / 17)		x					x
Woodall et al. (2014)	Both	DQ methods	By Friedman (2012)	16	(1 / 15)				x			
Barateiro and Galhardas (2005)	Researchers	DQ tools	By the authors	37	(9 / 28)				x		x	x
Pushkarev et al. (2010)	Researchers	Open-source tools	By the authors	6	(6 / 0)			x		x	x	x
Pulla et al. (2016)	Researchers	Open-source tools	By the authors	10	(10 / 0)			x		x	x	x
Gao et al. (2016)	Researchers	Big data DQ tools	By the authors	11	(4 / 7)						x	x
Ehrlinger and Wöß (This work)	Both	DQ tools	Systematic search	13	(5 / 8)		x	x	x	x		

### 3.2. Research Questions

The aim of this survey is to evaluate and compare existing DQ tools with respect to their DQ measurement and monitoring functionalities in order to answer the research question *how DQ measurement and monitoring concepts are implemented in state-of-the-art DQ tools*. This research question can be refined with three sub-questions, where the theoretical background is discussed in Section 2. In Section 4.1, we present our requirements catalog, in which each sub-question is assigned to specific technical requirements.

1. Which data profiling capabilities are supported by current DQ tools?
2. Which data quality dimensions and metrics can be measured with current DQ tools?
3. Do DQ tools allow automated data quality monitoring over time?

### 3.3. DQ Tool Search Strategy

To establish a comprehensive list of existing DQ tools, we developed a three-fold strategy. First, we included all observed tools from previous surveys by Barateiro and Galhardas (2005), Kokemüller and Haupt (2012), Gao et al. (2016), Selvage et al. (2017), Pulla et al. (2016), and Pushkarev et al. (2010) as candidate tools. Second, we conducted a systematic search to find research papers that introduce, investigate, or mention DQ tools. The third part of our search strategy consists of a random Google search by using the same search term combinations as for the systematic search. In contrast to the systematic search, we do not aim at a comprehensive observation of all search results, which is unfeasible for Google search results. However, to also identify non-research tools that have not been described in scientific papers, we consider this random search as enrichment to guarantee a best possible coverage of candidate tools. The remainder of this section is dedicated to the systematic search.

We identified the following search terms to conduct the systematic search: *data quality, information quality*, and *tool*. Since “information quality” is considered a synonym to “data quality” (Zhu et al., 2014), we applied both search terms to achieve higher coverage. We decided not to add the terms “assessment” and “monitoring” to the search, as it would automatically exclude tools that do not specifically use these keywords. Consequently, the following search expression has been applied:

(“*data quality*” ∨ “*information quality*”) ∧ *tool*

The search expression has then been applied to the list of digital libraries that is provided in Table 2. We also included the software development platform GitHub, because the purpose of this search is to select concrete tools. The original aim was to search all titles and abstracts from the computer science domain. However, since each digital library offers different search functionalities, we selected the closest search-engine-specific settings to reflect our original search aim. Table 2 documents the deviations for each conducted search along with the ultimately utilized search expression, which is already formatted according to the guidelines of the respective search engine. For the GitHub search, we additionally omitted the search term *tool*, because most GitHub results are obviously tools (except for empty repositories, code samples, or documentations).

**Table 2:** Systematic search.

**Source**	**Search expression**	**Scope**	**Restrictions**
ACM Digital Library^a^	acmdlTitle:(+(“data quality” “information quality”) +tool) OR recordAbstract:(+(“data quality” “information quality”) +tool)	Title, abstract	-
GitHub^b^	“data quality” OR “information quality”	Full text	-
Google Scholar^c^	allintitle: (“data quality” OR “information quality”) AND tool	Title	Exclude citations and patents
IEEE Xplore Digital Library^d^	(((“data quality”) OR “information quality”) AND tool)	Title, abstract, indexing terms	-
Science Direct^e^	TITLE-ABSTR-KEY(“data quality” OR “information quality”) and TITLE-ABSTR-KEY(tool)[All Sources(Computer Science)].	Title, abstract, keywords	Computer science only
Springer Link^f^	tool NEAR (“data quality” OR “information quality”)	Full text	Computer science only

a *http://dl.acm.org/advsearch.cfm (January, 2022)*.

b *https://github.com (January, 2022)*.

c *https://scholar.google.at (January, 2022)*.

d *http://ieeexplore.ieee.org/search/advsearch.jsp (January, 2022)*.

e *http://www.sciencedirect.com (January, 2022)*.

f *https://link.springer.com/advanced-search (January, 2022)*.

**Figure 1:**
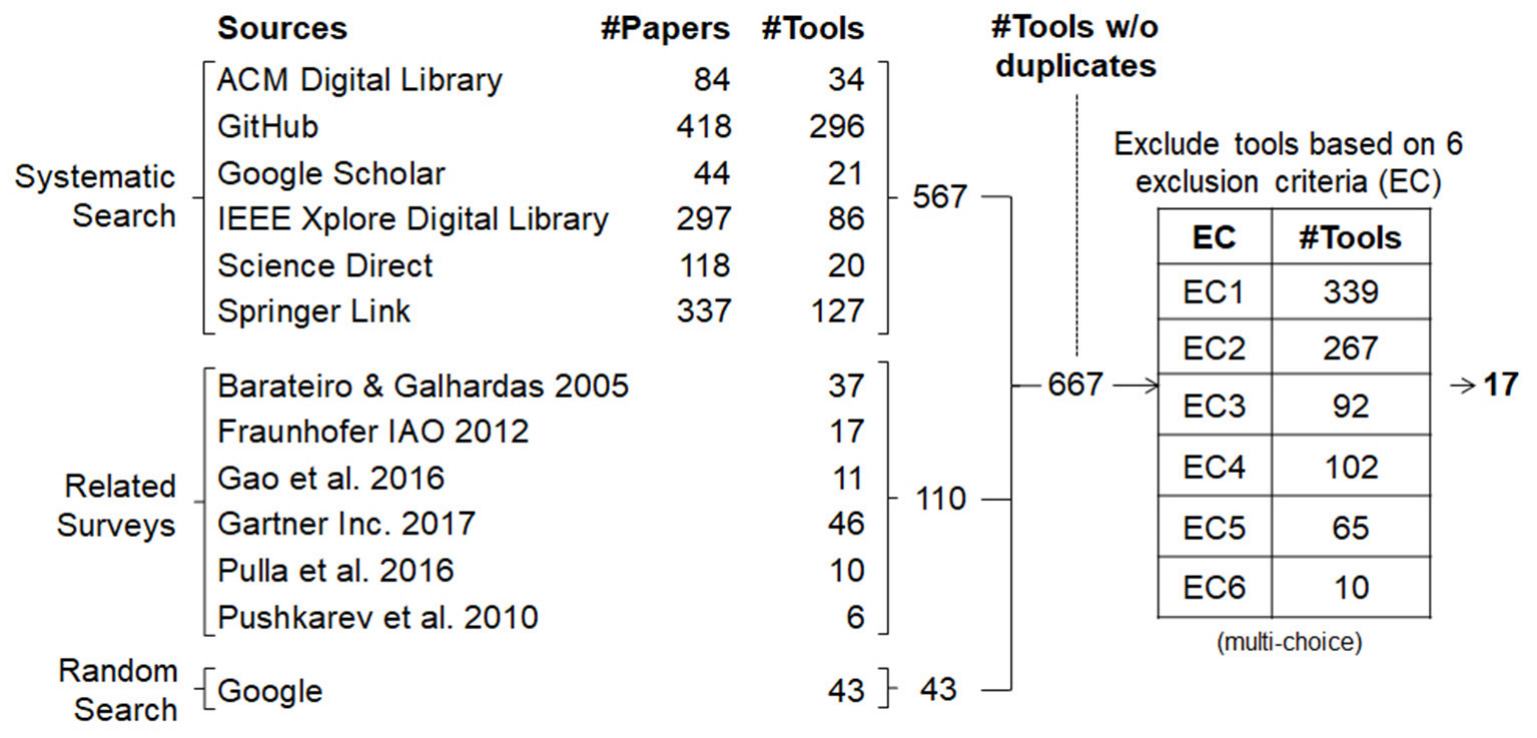
Systematic search.

For each search result, we assessed the title and abstract to determine whether a paper actually promotes a candidate DQ tool or not. In cases where title and abstract were not explicit enough, or they indicated the presentation of a tool (and therefore this article could not be directly classified as not relevant), the content of this article was investigated in more detail to record name and purpose of the tool in a first step. In the GitHub search, we excluded all tools that did not offer any kind of description immediately and used the others as candidates. Figure 1 illustrates the number of investigated research papers and the resulting tools. The next section describes the subsequent investigation of all candidate tools according to defined exclusion criteria (EC).

### 3.4. DQ Tool Selection

In accordance with our general search strategy, we defined three inclusion criteria. Each tool that was selected as candidate tool had to satisfy at least one of the following three criteria.

1. The tool was included in one of the previous surveys (cf. Barateiro and Galhardas, 2005; Pushkarev et al., 2010; Kokemüller and Haupt, 2012; Gao et al., 2016; Pulla et al., 2016; Selvage et al., 2017).
2. The tool was identified in our systematic search.
3. The tool was identified in our random search.

Figure 1 shows the number of scientific papers (#Papers), which we found in the systematic search per source, as well as the number of tools (#Tools) that were mentioned in these papers. It can be seen that some papers mention several DQ tools (e.g., other DQ tool surveys), while some use the term in their title or abstract, but do not refer to a concrete tool directly. In total, 1,298 papers have been discovered through the systematic search, which refer to 567 DQ tools (this number includes duplicates). In the related surveys we located 110 tools (including duplicates) and added 43 additional tools from the random Google search. In the next step, all 720 tools were merged into one file to remove duplicates. This resulted in a total of 667 identified distinct DQ tools. After establishing the list of candidate tools, we conducted a review to exclude all tools from the survey that met at least one of the following exclusion criteria.

(EC1) The tool is domain-specific (e.g., for web data or a specific implementations only).
(EC2) The tool is dedicated to specific data management tasks without explicitly offering DQ measurement.
  (a) The tool is dedicated to data cleansing.
  (b) The tool is dedicated to data integration (including on-the-fly DQ checks).
  (c) The tool is dedicated to other data management tasks (e.g., data visualization).
(EC3) The tool is not publicly available (e.g., the tool is only described in a research paper).
(EC4) The tool is considered deprecated (i.e., the vendor does not exist any more or the tool was found on GitHub and the last commit was before January 1^st^, 2016).
(EC5) The tool was found on GitHub without any further information available.
(EC6) The tool requires a fee and no free trial is offered upon request.

The table in Figure 1 shows how many tools were excluded per criterion (multiple selection was possible). Most of the tools were excluded because they are domain specific (EC1) and/or focus on specific data management tasks (EC2). The 267 tools excluded due to EC2 are divided between the three subcriteria as follows: 111 tools were excluded by EC2(a), 46 tools were excluded by EC2(b) and 110 tools by EC2(c).

For the search process and selection, we used Microsoft Excel to collect the identified scientific papers from the search engine results, to assemble a uniform list of identified DQ tools, and to remove duplicate tools. We tracked the exclusion of the tools according to our six criteria in a separate Excel file. 17 DQ tools have been selected for deeper investigation, from which 13 could be evaluated since three were based on SAP, where no installation was available, and one (IBM InfoSphere Information Server) could not be installed successfully during the time of the project, despite great effort but with little support from IBM.

### 3.5. Limitations of This Study

As pointed out by Pateli and Giaglis (2004), “the selection phase is critical, since decisions made at this stage undoubtedly have a considerable impact on the validity of the literature review results.” For our survey, we consider the conduction of the selection process and consequently its inherent limitations (cf. Kitchenham et al., 2009) as main threats to its validity. In this section, we specifically discuss the comprehension of our tool search strategy and the stringency of the exclusion criteria.

The following measures mitigated the risk of missing an important research paper and subsequently a DQ tool: (1) we used the online search engine Google Scholar in addition to the main publisher websites, (2) we specifically observed references from existing DQ tool surveys, and (3) we included a manual Google search in parallel to the systematic search.

Considering the ratio between the number of DQ tools selected for deeper investigation and the total number of identified DQ tools (17/667), the exclusion criteria might seem very stringent. We argue that they have been selected adequately for this survey due to the following reasons. First, we want to point out that there is a huge number of DQ tools (especially a subset of the 296 found on GitHub), which are only simple scripts to clean specific data sets. Two examples are SQL-Utils^1^, which consists of five SQL scripts for cleaning data and performing simple DQ checks, and DescribeCol^2^, which consists of one Python function that implements DQ tests by describing and visualizing a Pandas DataFrame. Although the dedicated investigation of domain specific DQ tools (EC1) is interesting future work, a further restriction of these tools would be required to compile meaningful results for such a study. Second, we deliberately excluded DQ tools that are restricted to specific data management tasks (e.g., data cleansing), because they do not support the answer of our general research question *how DQ measurement and monitoring concepts are implemented in state-of-the-art DQ tools*. Third, the time invested for each tool was about one person per month per tool. This was on the one hand due to the detailed requirements catalog (cf. Table 3), and on the other hand, for some tools already the installation or the negotiation with the customer support (e.g., to receive a full functional trial license) was very time-consuming. Considering this time effort, the investigation of all 667 DQ tools, or even only the 339 domain-specific tools, would be out of scope to answer our research question. Fourth, the number of selected DQ tools seems reasonable compared to related surveys. Investigating a considerable larger number of DQ tools would require the refinement of the entire evaluation process.

**Table 3:** DQ tool requirements catalog.

**Category**	**Sub-category**	**Requirement**
Data Profiling (Abedjan et al., 2015, 2019)	SC – Cardinalities	(1) “Number of rows” (Abedjan et al., 2019)
		(2) Number of null values (Abedjan et al., 2019)
		(3) “Percentage of null values” (Abedjan et al., 2019)
		(4) “Number of distinct values; sometimes called ‘cardinality' ” (Abedjan et al., 2019)
		(5) “Number of distinct values divided by the number of rows” (Abedjan et al., 2019)
	SC - Valuedistributions	(6) “Frequency histograms (equi-width, equi-depth, etc.)” (Abedjan et al., 2019)
		(7) “Minimum and maximum values in a numeric column” (Abedjan et al., 2019)
		(8) “Constancy: frequency of most frequent value divided by number of rows” (Abedjan et al., 2019)
		(9) “Quartiles: 3 points that divide the (numeric) values into 4 equal groups” (Abedjan et al., 2019)
		(10) “Distribution of first digit in numeric values; to check Benford's law” (Abedjan et al., 2019)
	SC - Patterns, data types, and domains	(11) “Basic type (numeric, alphanumeric, date, time, etc.)” (Abedjan et al., 2019)
		(12) “DBMS-specific data type (varchar, timestamp, etc.)” (Abedjan et al., 2019)
		(13) Measurement of value length (minimum, maximum, average, and median) (Abedjan et al., 2019)
		(14) “Maximum number of digits in numeric values” (Abedjan et al., 2019)
		(15) “Maximum number of decimals in numeric values” (Abedjan et al., 2019)
		(16) “Histogram of value patterns (Aa9...)” (Abedjan et al., 2019)
		(17) “Generic semantic data type” (Abedjan et al., 2019) [e.g., “code, date/time, quantity, identifier” (Abedjan et al., 2019)]
		(18) “Semantic domain” (Abedjan et al., 2019) (e.g., credit card, first name, city) (Abedjan et al., 2019)
	Dependencies	(19) “Unique column combinations” (Abedjan et al., 2019) (key discovery)
		(20) “Relaxed unqiue column combinations” (Abedjan et al., 2019)
		(21) “Inclusion dependencies” (Abedjan et al., 2019) (foreign key discovery)
		(22) “Relaxed inclusion dependencies” (Abedjan et al., 2019)
		(23) “Functional dependencies” (Abedjan et al., 2019)
		(24) “Relaxed functional dependencies” (Abedjan et al., 2019)
	Advanced MC profiling	(25) Correlation analysis (Abedjan et al., 2015)
		(26) Association rule mining (Abedjan et al., 2015)
		(27) Cluster analysis (Abedjan et al., 2015)
		(28) Outlier detection (Abedjan et al., 2015)
		(29) Exact duplicate tuple detection
		(30) Relaxed duplicate tuple detection
Data Quality Measurement	DQ Dimensions	(31) Metric to measure accuracy
		(32) Metric to measure completeness
		(33) Metric to measure consistency
		(34) Metric to measure timeliness
		(35) Metrics to measure other DQ dimensions
	Rule-based checks	(36) Creation of business rules
		(37) Availability of general-applicable integrity rules
		(38) Verification of data against business rules
Automated Data Quality Monitoring (Ehrlinger and Wöß, 2017)		(39) Scheduling a DQ metric or data profiling task in user-defined periods (Ehrlinger and Wöß, 2017)
		(40) Storage of DQ measurements and data profiling results (Ehrlinger and Wöß, 2017)
		(41) Retrieval of DQ measurements or data profiling results (Ehrlinger and Wöß, 2017)
		(42) Comparison between several DQ measurements or data profiling results (Ehrlinger and Wöß, 2017)
		(43) Visualization of DQ measurements / data profiling results over time (Ehrlinger and Wöß, 2017)

## 4. Design of the Evaluation Process

As outlined in Section 2.3, existing requirement frameworks for DQ tools did not adequately answer our research question. Thus, we developed a new catalog of requirements for the evaluation of DQ measurement and monitoring tools, which is discussed in the following subsection. The aim is to rate the fulfillment of each requirement with three categories: (✓) for fulfilled, (−) for not fulfilled, and (*p*) for partially fulfilled. In Section 4.2, we discuss the database used for the evaluation of the requirements and in Section 4.3 we list the predefined test cases to compare specific results between the investigated DQ tools.

### 4.1. Evaluation Requirements Catalog

Our requirements catalog in Table 3 consists of three main categories: data profiling (DP), data quality measurement (DQM), and continuous data quality monitoring (CDQM). The requirements for data profiling are based on the classification of DP tasks by Abedjan et al., which has been originally published by Abedjan et al. (2015), and updated by Abedjan et al. (2019). Since we started our survey prior to the classification update, our requirements catalog constitutes a tradeoff between the two versions. Since both versions contain the two sub-categories “single columns (SC) profiling” and “dependency detection,” we adhere here to the newer version by Abedjan et al. (2019). In the SC sub-category, we split the null values task (i.e., number or percentage of null values) in two different requirements: (DP-2) number of null values and (DP-3) percentage of null values, to separate the results. The newer version (Abedjan et al., 2019) contains an additional sub-category “metadata for non-relational data,” which is not included in our survey, because the evaluation for some tools with a fixed-period trial version was already completed at the time of the update. However, the original version (Abedjan et al., 2015) included a category “multi-column (MC) profiling,” which has been removed by Abedjan et al. (2019). We renamed this category to “advanced MC profiling” and added it along with two additional requirements (exact and relaxed duplicate tuple detection) to the end of the DP category. One reason for the exclusion of the MC sub-category from the data profiling task taxonomy by Abedjan et al. (2019) might be the strong overlap of these tasks with the field of data mining. Abedjan et al. (2019) point out that there exists no clearly defined and widely accepted distinction between the two research fields. Thus, although a separate category for those requirements could be argued, we decided to include it in the data profiling category, because data mining is not in the focus of our survey.

The category for DQ measurement contains requirements to provide metrics for specific DQ dimensions and business rule management capabilities. While we listed metrics for the DQ dimensions accuracy, completeness, consistency, and timeliness as described in Section 2.2 explicitly, we investigate the existence of additional metrics during our evaluation by means of (DQM-34). Since DQ dimensions such as consistency are often measured with a set of rules (cf. Section 2.2.3) and the development of business rules is generally regarded as the basis for DQ measurement in some methodologies (cf. Sebastian-Coleman, 2013), we have expanded our catalog to include (DQM 35-37). It is distinguished between the (DQM-35) creation of domain-specific business rules and the (DQM-36) availability of general integrity rules, for example a birth date cannot be in the future or a temperature value can never reach -270 °C. It should also be possible to verify those rules (DQM-37).

The requirements for CDQM are based on the findings from our previous research published by Ehrlinger and Wöß (2017) and summarize key tasks to ensure automated DQ monitoring over time. The continuous measurement, storage, and usage of the collected metadata should be possible for both data profiling results and DQ measurements.

### 4.2. Evaluation Database

**Figure 2:**
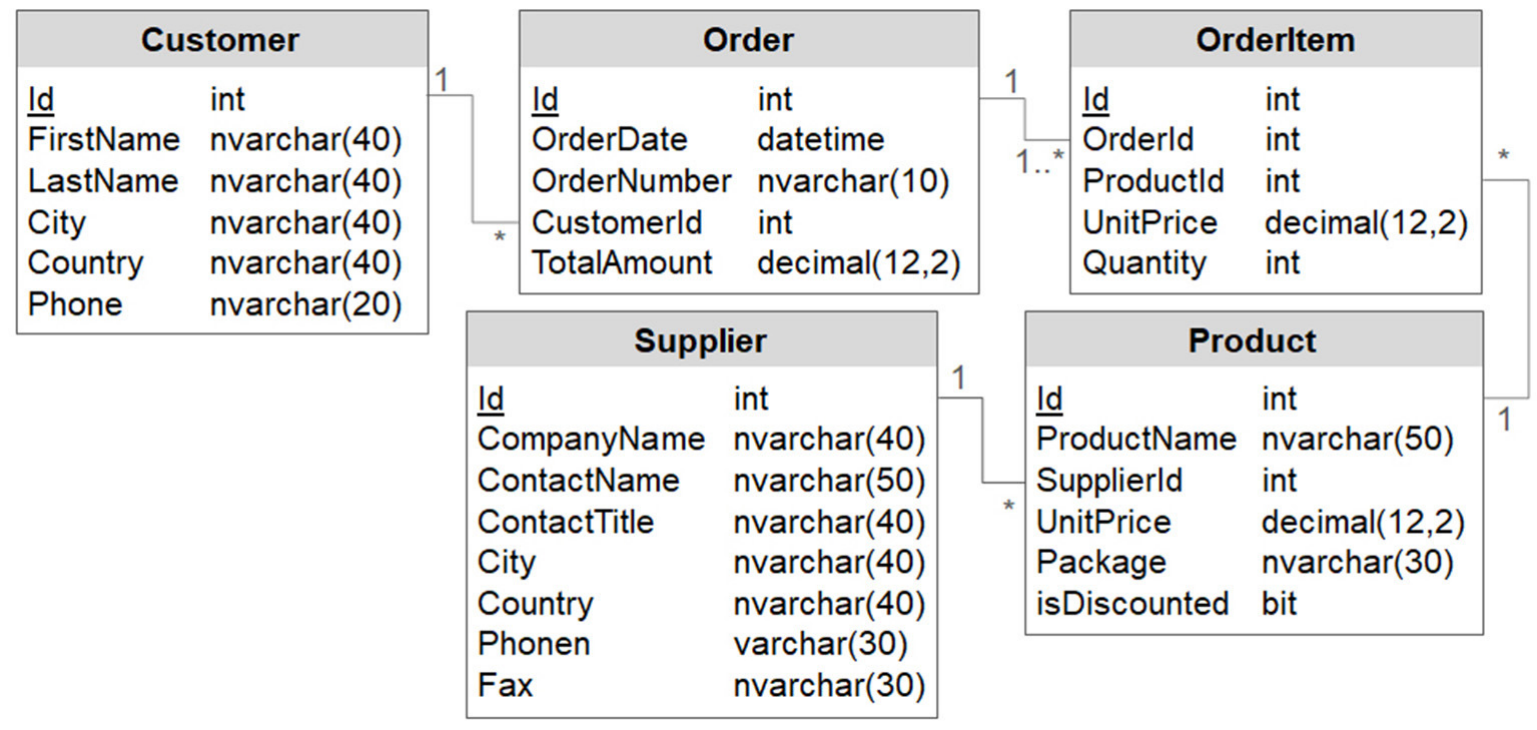
Schema of the northwind evaluation DB.

For the evaluation of the requirements from Table 3, we used a modernized version of the well-known Northwind DB published by dofactory^3^. Figure 2 illustrates the schema of the database with five tables as UML (unified modeling language) class diagram. Foreign key relationships and their cardinalities are represented in UML notation.

### 4.3. Data Profiling Test Cases

To compare the results of the requirements between the DQ tools, we defined a test case for each requirement from the data profiling category. We did not define such fine-grained test cases for the DQ measurement category since the DQ metric implementations were too diverse to compare their results directly. Also, the requirements of the DQ monitoring category do not yield a comparable result (e.g., in form of numbers), and hence there are no test cases. The following list comprises all test cases we performed for the DP category, whereby the enumeration can be linked to the DP requirements from Table 3:

1. Number of rows in table Product.
2. Number of nullvalues in column Supplier.Fax.
3. Percentage of nullvalues in column Supplier.Fax.
4. Number of distinct values in column Customer.Country.
5. Number of distinct values divided through number of rows for Customer.Country.
6. Frequency histograms for Customer.Country.
7. Minimum and maximum values in OrderItem.UnitPrice.
8. Constancy for column Customer.Country.
9. Quartiles in column OrderItem.UnitPrice.
10. Distribution if first digit=1 in column UnitPrice, table OrderItem.
11. Basic types for ProductName, UnitPrice, and isDiscontinued in table Product.
12. DBMS-specific data types for ProductName, UnitPrice, and isDiscontinued in table Product.
13. Minimum, maximum, average, and median value length of column Product.ProductName.
14. Maximum number of digits in column Product.UnitPrice.
15. Maximum number of decimals in column Product.UnitPrice.
16. Count of pattern “AA” in Customer.Country, derived from histogram
17. Semantic data types for ProductName, UnitPrice, and isDiscontinuedin table Product.
18. Semantic domains for ProductName, UnitPrice, and isDiscontinuedin table Product.
19. All 100 % conforming UCCs in Order.
20. All 98 % conforming UCCs in Order.
21. All 100 % conforming INDs between Order.CustomerIdand Customer.Id.
22. All 93 % conforming INDs between Order.CustomerIdand Customer.Id.
23. All 100 % conforming FDs in Order.
24. All 93 % conforming FDs in Order.
25. Correlation between OrderItem.UnitPriceand OrderItem.Quantity.
26. All possible association rules within Product.
27. Clustering the values in Product.UnitPrices.
28. All “very high values” in Order.TotalAmount.
29. All exact duplicates in Customer, considering FirstNameand LastNameonly.
30. All relaxed duplicates in Customer, considering FirstNameand LastNameonly.

All test cases were conducted by two researchers (one of whom is the lead author of this article), who verified each other's results.

## 5. Data Quality Tool Evaluation

In this section, we first describe the DQ tools, which we selected for the evaluation, and second, we investigate the selected tools with respect to our evaluation framework and discuss the requirements.

### 5.1. Selected Data Quality Tools

In total, we selected 17 DQ tools for detailed evaluation. Three of them were based on SAP (SAP Information Steward, DQ solution by ISO Professional Services, and dspCompose by BackOffice Associates GmbH) and since we had no access to a SAP installation, we did not include these tools in our survey, but described them textually. To achieve a comparable overview on the investigated DQ tools, we formulated the following seven questions.

- Which exact version did we evaluate? (DQ tool name and version).
- Who is the vendor or creator of the tool?
- Is the tool open-source?
- How did we perceive the user interface? (1–5 rating, 5 is best).
- How did we perceive customer support? (1–5 rating, 5 is best).
- How was the investigated DQ tool provided? (e.g., freely available on GitHub/SourceForge or trial license).
- In which scientific paper or on which online platform was the DQ tool found?

An overview on the answers to the questions is given in Table 4 and a detailed discussion is provided in the following subsections (DQ tools listed in alphabetical order). Since the focus of this survey is on the measurement functionality of DQ tools, technical details like the adoption (i.e., on-premise vs. SaaS) was not relevant for answering our research questions. We refer to related surveys for more technical details, especially the Gartner Magic Quadrant (cf. Chien and Jain, 2019) and Fraunhofer IAO (cf. Kokemüller and Haupt, 2012).

**Table 4:** Summary of investigated DQ tools.

**DQ Tool Version**	**Vendor / Creator**	**Open** **-source**	**User** **Interface**	**Customer** **Support**	**Provided**	**Found With**
Aggregate Profiler 6.2.4	Arrah Technology	Yes	2 (out of 5)	Not consulted	Free on SourceForge	Dai et al., 2016
Apache Griffin 0.2.0	Apache Foundation	Yes	4 (out of 5)	Not available	Free on GitHub	GitHub
Ataccama ONE profiler	Ataccama	No	5 (out of 5)	1 (out of 5)	Free online version	Pushkarev et al., 2010; Kokemüller and Haupt, 2012; Abedjan et al., 2015; Judah et al., 2016; Pulla et al., 2016; Selvage et al., 2017
DataCleaner Enterprise Edition 6.3.0	Human Inference	No	5 (out of 5)	5 (out of 5)	Full trial (3 month)	Pushkarev et al., 2010; Gao et al., 2016; Pulla et al., 2016; Selvage et al., 2017
Datamartist 1.7.9	nModal Solutions	No	2 (out of 5)	Not consulted	Full trial (30 days)	Pulla et al., 2016
Pandora 5.9.0	Experian	No	4 (out of 5)	5 (out of 5)	Full trial (30 days)	Judah et al., 2016; Selvage et al., 2017
Informatica Data Quality 10.2.0	Informatica	No	4 (out of 5)	5 (out of 5)	Full trial (2 x 30 days)	Abedjan et al., 2015; Gao et al., 2016; Judah et al., 2016; Selvage et al., 2017
InfoSphere Information Server for Data Quality 11.7	IBM	No	Not evaluated	1 (out of 5)	Full trial (3 month)	Kokemüller and Haupt, 2012; Abedjan et al., 2015; Judah et al., 2016; Selvage et al., 2017
InfoZoom Desktop Professional 2018 release 9.20 and IZDQ 2018.03	humanIT Software GmbH	No	4 (out of 5)	4 (out of 5)	Full trial (6 month)	Kokemüller and Haupt, 2012
MobyDQ, pulled 05/21/19	Alexis Rolland	Yes	3 (out of 5)	4 (out of 5)	Free on GitHub	GitHub
OpenRefine version 3.0 and MetricDoc extension (pulled on Feb. 14th 2019)	-	Yes	2 (out of 5)	Not available	Free on GitHub	Tsiflidou and Manouselis, 2013; Kusumasari et al., 2016, GitHub
Enterprise Data Quality pre-built Virtual Machine 12.2.1	Oracle	No	3 (out of 5)	Not consulted	Free on Oracle website	Abedjan et al., 2015; Judah et al., 2016; Selvage et al., 2017
Open Studio for Data Quality 6.5.1	Talend	Yes	4 (out of 5)	2 (out of 5)	Free on GitHub / Talend website	Pushkarev et al., 2010; Gao et al., 2016; Judah et al., 2016; Pulla et al., 2016; Selvage et al., 2017, GitHub
SAS Base 9.4 and SAS Data Quality Desktop 2.7	SAS	No	3 (out of 5)	3 (out of 5)	Full trial (60 days)	Barateiro and Galhardas, 2005; Maletic and Marcus, 2009; Judah et al., 2016; Selvage et al., 2017

#### 5.1.1. Aggregate Profiler

Aggregate Profiler (AP) is a freely available DQ tool, which is dedicated to data profiling. The tool was discovered twice in our systematic search: once because it was mentioned by Dai et al. (2016) in the Springer search results, and once in the Google search results, since it is also published on Sourceforge as “Open Source Data Quality and Profiling,”^4^ developed by *arrah* and *arunwizz*. In addition to its data profiling capabilities, like statistical analysis and pattern matching, Aggregate Profiler can also be used for data preparation and cleansing activities, like address correction or duplicate removal. Moreover, business rules can be defined and scheduled in user-defined periods. We perceived the user interface (UI) as inferior compared to other tools, since the navigation and application of DP functions was not intuitive.

#### 5.1.2. Apache Griffin

Apache Griffin^5^ (AG) differs significantly from the other tools in this survey, because it does not offer any data profiling functionality and is not a comprehensive DQ solution. However, since part of the evaluation is to observe the extent to which current tools support CDQM, we included Apache Griffin, since it is dedicated to continuously measure the quality of Big Data, both batch-based and streaming data. We installed Apache Griffin 0.2.0, which is still in the incubator status of Apache, on Ubuntu 18.04. The tool requires the following dependencies, from which some are (at the time of the installation) still in incubating status as well: JDK (1.8+), MySQL DB, npm, Hadoop (2.6.0+), Spark (2.2.1+), Hive (2.2.0), Livy, and ElasticSearch. Due to these dependencies, the installation was very cumbersome in contrast to other tools. In our case, two experienced computer scientists needed over a week to complete the full installation. Once installed, the UI is intuitive and supports the domain-specific definition of accuracy metrics as well as the scheduling and monitoring of those metrics. Other DQ metrics, like completeness, are planned to be integrated in future versions.

#### 5.1.3. Ataccama ONE

The company Ataccama with its headquarters in Canada offers several DQ products, which we found through different sources in our search: Data Quality Center and Master Data Center have been previously investigated by Kokemüller and Haupt (2012); DQ Analyzer has been included in Pushkarev et al. (2010) and Pulla et al. (2016) and in Abedjan et al. (2015). Gartner additionally mentioned the DQ Issue Tracker and the DQ Dashboard in 2016 (Judah et al., 2016). However, since 2017, Ataccama consolidated their separate DQ solutions into “Ataccama ONE” (A-ONE). While the license of the full DQ solutions is subject to costs, the data profiling module of Ataccama ONE can be accessed freely. Unfortunately, Ataccama customer support did not provide us with a trial license of the complete ONE solution. Thus, we were only able to investigate the free “Ataccama ONE profiler,”^6^ where the focus is on data profiling and which does not provide monitoring functionality. We performed the evaluation of the online-available tool during October 2018. According to Gartner (cf. Selvage et al., 2017) and Ataccama customer support, the full solution would provide a much richer scope of functions, including DQ monitoring, but we were not able to investigate it. The data profiling module was very intuitive and easy to use, also for business users. In terms of customer support (from Prague), we experienced very long response times on our contact attempts for a license request. Additionally, we were promised to receive a training as prerequisite to test the full Ataccama ONE solution, which was never redeemed due to the workload on the side of Ataccama.

#### 5.1.4. DataCleaner by Human Inference

The DQ products “DataCleaner” (DC) and “DataHub” were originally developed by Human Inference, which was incorporated into Neopost in 2012, later into Quadient, and since 2019 into the EDM Media Group, where it is again promoted with its original name “Human Inference.” DataCleaner offers dedicated and independent DQ measurement functionality, although pure data cleansing functions might be expected due to its name. Our customer contact declared that the professional version of DataCleaner (in contrast to the community edition that is freely available on GitHub) offers the same DQ measurement functionalities as DataHub, but differs only with respect to the convenient usage, the UI, and the data integration features. Thus, we evaluated a full trial of DataCleaner Enterprise Edition, which aims at people with technical background. In addition, we were able to observe the functionalities of DataHub in an interactive web session. Human Inference places emphasis on customer data, which is reflected in special algorithms for duplicate detection, address matching, and data cleansing. Under the vendor Quadient, DataCleaner was mentioned by in Selvage et al. (2017) (Gartner Inc.), but excluded from the follow-up survey by Chien and Jain (2019) due to strategic changes. DataCleaner was previously observed by Pushkarev et al. (2010); Gao et al. (2016); Pulla et al. (2016), but under different vendors. Although DataCleaner is built for technical users, we perceived the UI as very intuitive. DataHub (with its vision of a single customer view) offers in addition to the administrator's view a data steward view, which is specifically dedicated to business users, for example, to resolve ambiguous duplicates. We also want to highlight [in conformance with Selvage et al. (2017)] the very helpful and friendly customer support that provided us with the trial license and more insight in DataHub.

#### 5.1.5. Datamartist by nModal Solutions Inc.

The commercial tool Datamartist^7^ (DM) by nModal Solutions Inc. requires the operating system Microsoft Windows and the .NET framework 2.0 to be installed. Datamartist is dedicated to data profiling and data transformation. The investigated 30-days trial offers all Pro edition features. Since the trial could be downloaded from the website directly, we did not consult any customer support. We perceived the UI of Datamartist as slightly inferior compared to other commercial tools since for some tasks (e.g., exporting data profiling results) the command line was required.

#### 5.1.6. Experian Pandora

The company Experian with its headquarters in Ireland offers two commercial DQ solutions: Cleanse and Pandora (EP). During the conduct of our survey, they introduced the new product Aperture Data Studio, which is going to replace Pandora in the future. While Cleanse is dedicated to one-time-data-cleansing, we investigated the more comprehensive tool Pandora. In accordance with the findings by Selvage et al. (2017) (Gartner Inc.), we perceived the tool as easy to install and use and want to highlight the comprehensive data profiling capabilities in general, and the cross-table profiling capabilities in particular. In addition, Pandora provides a rich ability to extend the existing feature palette with customized functions. In summary, Pandora achieved one of the best overall assessments in our survey. We perceived the UI as good, though more dedicated to technical users, and had very good experience with the technical customer support who supported us in a timely and target-oriented fashion.

#### 5.1.7. Informatica Data Quality

Informatica Data Quality (IDQ) is one module of the commercial data management solution by Informatica, which is according to Gartner (cf. Judah et al., 2016; Selvage et al., 2017; Chien and Jain, 2019), leader in the Magic Quadrant of Data Quality Tools for several years. We were provided with two 30-days trial licenses. The trial included the Informatica Developer (the desktop installation for developers), Informatica Analyst (the web-based platform for business users), and Informatica Administrator (for task scheduling), where all three user interfaces access the same server-side backend of Informatica DQ version 10.2.0. In our systematic search, we found five different tools offered by the company Informatica, from which four had been excluded from the evaluation. For example, the “Master Data Management” solution was excluded due to the focus on master data management. Informatica Data Quality was found through the Springer Link search (cf. Abedjan et al., 2015), and because it was previously investigated by Judah et al. (2016); Selvage et al. (2017), and Gao et al. (2016). Informatica has its origin in the field of data integration and in addition to the features we evaluated, they offer data cleansing and matching functionalities. In terms of DQ measurement, they offer most probably the closest implementation to the DQ dimension and metric view promoted in the research community. We perceived the UI of Informatica Analyst as easy to use, also for business users, but with less comprehensive functionality than the Informatica Developer, which is more powerful and dedicated to trained and technical users. In accordance to the findings by Gartner customers (cf. Selvage et al., 2017), we can confirm the very helpful sales support, which was one of the best we experienced. During the evaluation, we had regular web conferences to ask questions and review the results, and short intermediate requests were answered timely.

#### 5.1.8. IBM InfoSphere Information Server for Data Quality

The product “Infosphere Information Server for Data Quality” (IBM ISDQ) by IBM was found through the studies by Gartner (cf. Judah et al., 2016; Selvage et al., 2017) and Fraunhofer IAO (cf. Kokemüller and Haupt, 2012). Other product (or product components) from IBM have also been previously mentioned in the following research papers: IBM Informix (previously called “DataBlade”) by Barateiro and Galhardas (2005), IBM InfoSphere Information Analyzer by Abedjan et al. (2015), IBM QuerySurge by Gao et al. (2016), IBM Data Integrator by Chen et al. (2012), IBM InfoSphere MDM Server by Pawluk (2010), and IBM Quality Stage by Prasad et al. (2011). For our survey, the IBM partner solvistas GmbH, located in Austria, provided us with the installation files of IBM InfoSphere Information Server for Data Quality version 11.7 for a three-month trial. Unfortunately, we were not able to evaluate the tool due to an early error in the installation process stating that a required file was not found. Despite intensive research of the documentation^8^, it was not possible to resolve the issue within the timeframe of the project, since no support by IBM nor any specific installation instruction for the received files was provided. We also contacted Fraunhofer IAO, who included IBM ISDQ in their survey (Kokemüller and Haupt, 2012). However, they did not install the tool, but based their statements on contact with the IBM support and the documentation. Also solvistas GmbH claimed that, so far, they never installed the IBM DQ product line. This experience aligns with the statement by Gartner that reference customer rate the technical support and documentation of IBM below the average (Chien and Jain, 2019).

#### 5.1.9. InfoZoom by humanIT Software GmbH

InfoZoom is a commercial DQ tool by the German vendor humanIT Software GmbH^9^ and is dedicated to data profiling using in-memory analytics. It was previously surveyed by Kokemüller and Haupt (2012). We investigated InfoZoom Desktop Professional with the IZDQ (InfoZoom Data Quality) extension in a 6-month license granted to us from the customer support. While InfoZoom Desktop is dedicated to data profiling and data investigation, the IZDQ extension allows a user to define rules and jobs for comprehensive DQ management. Generally, InfoZoom aims at observing and understanding the data but does not support any cleansing activities, which aligns well with the observations performed in this survey. We perceived the UI of InfoZoom Desktop as easy to use, also for business users, whereas the IZDQ extension requires technical knowledge like the ability to write SQL statements, or at least, intensive training to be used by non-technical users. The customer support was very friendly and helpful and provided us in a timely manner with a relatively long trial licenses in comparison to other commercial DQ tools.

#### 5.1.10. MobyDQ

MobyDQ^10^, which was previously termed “Data Quality Framework,” by Alexis Rolland is a free and open-source DQ solution that aims to automate DQ checks during data processing, storing DQ measurements and metric results, and triggering alerts in case of anomaly. The tool was inspired by an internal DQ project at Ubisoft Entertainment, which differs to the open-source version with respect to software dependency and mature but context-dependent configuration. We found MobyDQ through our GitHub search and evaluated the version downloaded on May 21nd, 2019. Similar to the commercial tools we observed, the framework can be used to access different data sources. In contrast to Apache Griffin, MobyDQ could be installed quickly and straightforward, based on the detailed documentation provided on GitHub. MobyDQ does not provide any DP functionality, because its focus is on the creation, application, and automation of DQ checks. The creator Alexis Rolland was very helpful in demonstrating the productive installation at Ubisoft Entertainment to us, which clearly demonstrates the potential of the tool when applied in practice.

#### 5.1.11. OpenRefine and MetricDoc

OpenRefine^11^ (formerly Google Refine, abbrev. OR) is a free and open-source DQ tool dedicated to data cleansing and data transformation and was discovered through (Kusumasari et al., 2016) in the IEEE search results, and (Tsiflidou and Manouselis, 2013) in the Springer Link search results as well as on GitHub^12^. While the original functionality of the tools does not primarily align with the focus of our survey, its extension MetricDoc specifically aims at assessing DQ with “customizable, reusable quality metrics in combination with immediate visual feedback” (Bors et al., 2018). Apart from the mention by Tsiflidou and Manouselis (2013) and Kusumasari et al. (2016), OpenRefine was not evaluated in one of the previous DQ tool surveys, although it is open source. We installed the tool from GitHub and evaluated OpenRefine version 3.0 with the MetricDoc extension (where no version was provided), downloaded on February 14th, 2019. We perceived the usability of OpenRefine as average and especially in the MetricDoc extension, the usability of several functions reflected its state as very current research project.

#### 5.1.12. Oracle Enterprise Data Quality

The commercial tool Oracle Enterprise Data Quality (EDQ) was previously mentioned by Gartner (cf. Judah et al., 2016; Selvage et al., 2017) and also found in the Springer Link search results (Abedjan et al., 2015). We investigated the freely available pre-built Virtual Machine available at the Oracle website^13^. In addition to classical data profiling capabilities, EDQ offers data cleansing (parsing, standardization, match and merge, address verification), as well as DQ monitoring to some extent. The GUI was perceived as average with the major drawback being the inflexible data source connection to DBs and files. In comparison to other DQ tools, where a connection can be directly accessed and reused, Oracle EDQ requires a “snapshot” of the actual data connection to be created prior to any profiling or DQ measurement task. This approach prevents an automatic update of the data source. We did not require contact to the customer support and the install documentation and user guide was up-to-date and very intuitive to use.

#### 5.1.13. Talend Open Studio for Data Quality

The company Talend offers two DQ products: Talend Open Studio (TOS) for Data Quality (a free version) and Talend Data Management Platform (requires subscription). Gartner upgraded Talend in their Magic Quadrant of Data Quality Tools from being “visionary” in 2016 to “leader” in 2017 (cf. Judah et al., 2016; Selvage et al., 2017). Talend Open Studio for Data Quality is one of the most frequently cited DQ tools that we discovered in our systematic search: it was found through Springer Link and GitHub^14^ and was already previously investigated by Pushkarev et al. (2010); Gao et al. (2016); Pulla et al. (2016). Both products (Open Studio and Enterprise) offer good support for Big Data analysis like Spark or Hadoop and a variety of data profiling and cleansing functionalities. We evaluated version 6.5.1 of TOS for Data Quality, which can definitely keep up with several commercial DQ tools (which require a fee) in terms of data profiling capabilities, business rule management, and UI experience. However, the free version does not support DQ monitoring capabilities, which is an exclusive feature of the Enterprise edition. It was not possible to receive a free trial of the Talend Data Management Platform, because according to our customer contact, it is unlikely that someone would purchase the Enterprise edition because of this feature.

#### 5.1.14. SAS Data Quality

The US company SAS^15^ (Statistical Analysis System) offers three commercial DQ products: SAS Data Management, SAS Data Quality, and SAS Data Quality Desktop (Chien and Jain, 2019). Since, the traditional focus of SAS is on data analysis, their DQ product is based on the acquired company DataFlux. The product “dfPower” by DataFlux has previously been surveyed by Barateiro and Galhardas (2005) and is mentioned by Maletic and Marcus (2009), which was discovered through our systematic search. In our evaluation, we did not find powerful machine learning (ML) capabilities (as core strength of SAS) in DQ measurement, which was also mentioned by Selvage et al. (2017). According to our customer contact and also mentioned by Chien and Jain (2019), SAS' overall strategic focus is on migrating all product lines into the cloud-based SAS Viya platform to increase the usability and to better integrate ML and DQ. In the evaluated tool SAS Data Quality Desktop 2.7, we found that the overall usability was below the average when compared to other DQ tools. The customer support was friendly, but hardly any question could be answered directly.

#### 5.1.15. Data Quality Solutions Dedicated to SAP

SAP (German abbreviation for “Systeme, Anwendungen und Produkte in der Datenverarbeitung,” i.e., “Systems, Applications and Products in Data Processing,”) is a worldwide operating company for enterprise application software with headquarters in Germany. Since SAP is market leader in the data processing domain, there are several DQ tools that are specifically built to operate on top of an existing SAP installation. During this survey, we had no access to such an installation, and thus, were not able to include those tools in our evaluation. However, due to the practical relevance of DQ measurement in SAP, we describe the most relevant tools dedicated to SAP, which we found through our systematic search.

##### 5.1.15.1. SAP Information Steward

SAP Information Steward was found through our systematic search and previously mentioned by Chien and Jain (2019); Abedjan et al. (2015, 2019). According to the documentation, the tool offers different data profiling functionalities (like simple statistics, histograms, data types, and dependencies), allows to define and execute business rules, as well as to monitor DQ with scorecards. Its strength are the wide range of out-of-the-box functions for specific domains like customers, supply chains, and products, however, customers often state that the costs for the product are too high and the interface needs some modernization for business users (Chien and Jain, 2019).

##### 5.1.15.2. Data Quality Solution by ISO Professional Services

The German company ISO Professional Services offers a data governance solution, which is implemented directly in SAP and reuses user-defined business rules from the SAP environment. A few years ago, ISO acquired the company Scarus Software GmbH with the DQ tool DataGovernanceSuite, which was discovered through our search and was previously evaluated by Kokemüller and Haupt (2012). The Scarus Data Quality (SDQ) Server constitutes the core DQ component by ISO, which has a separate memory but no DB. SDQ interoperates with SAP transparently, by offering functions like data profiling, duplicate detection, and address validation, which are directly executed within SAP. In contrast to its competing product SAP Information Steward, which aims at large enterprises, the tool by ISO is optimized for small to medium-sized companies. Reference customers of this size preferred the tool by ISO Professional Services due to its adjusted functional scope and cheaper pricing.

##### 5.1.15.3. dspCompose by BackOffice Associates GmbH

The German company BackOffice Associates GmbH offers a DQ suite prefixed with “dsp” (data stewardship platform), which is dedicated to master data management. Their primary DQ products are dspMonitor (for data profiling, monitoring, and DQ checks), which is a competing product to SAP Information Steward, and dspCompose (for data cleansing and DQ workflow management), which acts as add-on for dspMonitor or SAP Information Steward. Further DQ related products are dspMigrate, an end-to-end data migration tool, dspConduct, a SAP MDE tool, and dspArchive for data achiving in SAP environments. Although BackOffice Associates offer their DQ products to customers without SAP, they developed a strong SAP focus in recent years. According to our customer contact, they leverage the greatest potential in offering dspCompose in combination with SAP.

### 5.2. Comparison of Data Profiling, DQ Measurement, and Monitoring Capabilities

In this Section, we investigate the DQ tools with regard to our catalog of requirements from **Table 3**. For each requirement, three ratings are possible: (✓) the requirement is fulfilled, (−) the requirement is not fulfilled, or (*p*) the requirement is partially fulfilled. The coverage of each requirement is described in textual form with a focus on the justification of partial fulfillments.

#### 5.2.1. Data Profiling Capabilities

Table 5 shows the fulfillment of data profiling capabilities for each tool. We excluded Apache Griffin and MobyDQ from this table, because both tools do not offer any data profiling functionality. It can be summarized that basic single-column data profiling like cardinalities (DP 1–5) are covered by most tools, but more sophisticated functionalities, like dependency discovery and multi-column profiling, are offered only in single cases.

**Table 5:** Data profiling capabilities.

		**Aggregate Profiler**	**Ataccama ONE**	**DataCleaner**	**Datamartist**	**Experian Pandora**	**Informatica DQ**	**InfoZoom & IZDQ**	**OpenRefine & MetricDoc**	**Oracle EDQ**	**SAS Data Quality**	**Talend Open Studio**
1	Number of rows	✓	✓	✓	✓	✓	✓	✓	✓	✓	✓	✓
2	Number of nulls	✓	✓	✓	✓	✓	✓	✓	✓	✓	✓	✓
3	Percentage of nulls	−	✓	−	✓	✓	✓	✓	✓	✓	✓	✓
4	Number of distinct values	✓	✓	✓	✓	✓	✓	✓	✓	✓	✓	✓
5	Percentage of distinct values	−	✓	−	✓	✓	✓	✓	−	−	✓	✓
6	Frequency histograms	−	−	*p*	*p*	*p*	*p*	*p*	*p*	*p*	−	*p*
7	Minimum and maximum values	✓	✓	✓	✓	✓	✓	✓	−	✓	✓	✓
8	Constancy	−	✓	−	✓	✓	✓	✓	−	✓	✓	✓
9	Quartiles	✓	*p*	✓	−	−	−	*p*	−	−	*p*	✓
10	Distribution of first digit	−	−	−	−	−	−	−	−	−	−	✓
11	Basic types	−	✓	*p*	✓	✓	✓	✓	✓	✓	✓	−
12	DBMS-specific data type	✓	−	✓	−	−	✓	−	−	−	✓	✓
13	Value length	✓	✓	✓	−	✓	*p*	*p*	*p*	*p*	*p*	*p*
14	Number of digits	✓	−	✓	−	✓	✓	✓	−	✓	−	−
15	Number of decimals	✓	−	−	−	✓	✓	✓	−	−	−	−
16	Histogram of value patterns	−	−	✓	✓	✓	✓	✓	−	✓	*p*	✓
17	Generic semantic data type	−	−	✓	−	✓	✓	−	−	✓	✓	✓
18	Semantic domain	−	−	✓	−	✓	✓	−	−	✓	✓	✓
19	UCCs (key discovery)	−	−	−	−	*p*	✓	−	−	*p*	*p*	−
20	Relaxed UCCs	−	−	−	−	*p*	✓	−	−	−	−	−
21	INDs (foreign key discovery)	−	−	−	−	✓	*p*	−	−	−	*p*	−
22	Relaxed INDs	−	−	−	−	✓	*p*	−	−	−	*p*	−
23	FDs	−	−	−	−	✓	✓	−	−	−	−	*p*
24	Relaxed FDs	−	−	−	−	✓	✓	−	−	−	−	*p*
25	Correlation analysis	✓	−	−	−	−	−	−	−	−	−	*p*
26	Association rule mining	−	−	−	−	−	−	−	−	−	−	−
27	Cluster analysis	*p*	−	−	*p*	−	*p*	−	*p*	*p*	*p*	−
28	Outlier detection	*p*	*p*	−	*p*	✓	*p*	*p*	−	−	*p*	−
29	Exact duplicate detection	*p*	−	✓	*p*	✓	✓	✓	*p*	✓	✓	✓
30	Relaxed duplicate detection	*p*	−	✓	−	✓	✓	✓	*p*	✓	✓	✓

##### 5.2.1.1. Single Column—Cardinalities

While simple counts of values (i.e., cardinalities), like the number of rows, null values, or distinct values are covered by all DQ tools that support data profiling in general, the major distinction is an out-of-the-box availability of percentage values. The percentage of null values (DP-3) or distinct values (DP-5) is not supported by all investigated tools. The test case results also reveal different precision for the calculation of the percentages. For example, the percentage of null values in column Supplier.Fax was 55 % with Datamartist, 55.2 % with Oracle EDQ and SAS DataFlux, and 55.17 % in all other tools. The test case for DP-5 yielded 23 % with Datamartist, 23.07 % with Informatica, and 23.08 % with the other tools.

##### 5.2.1.2. Single Column—Value Distributions

Value distributions can be described as cardinalities of value groups (Abedjan et al., 2015). While histograms to visualize value distributions are available in most tools in the form of equi-width histograms (which “span value ranges of same length” Abedjan et al., 2015), we did not find any tool that supports equi-depth or equi-height histograms (where each bucket represents “the same number of value occurrences.” Thus, we rated all tools that support histograms with “partially” for DP-6. Ataccama allows frequency analysis but no visualization with histograms, and Aggregate Profiler visualizes the distributions only in form of a pie chart. The majority of tools also support minimum and maximum values (DP-7), as well as constancy (DP-8), which is defined as “the ratio of the frequency of the most frequent value (possibly a predefined default value) and the overall number of values” (Abedjan et al., 2015). “Benford's law” (DP-10), which is particularly interesting in the area of fraud detection, was only available in Talend OS.

Quantiles are a statistical measure to divide a value distribution into equidistant percentage points (Sheskin, 2003). The most common type of quantiles, which we observed in our study, are “quartiles” (DP-9), where the value distribution is divided by three points into four blocks. The division points are a multiple of 25 %, denoted as lower quartile or Q1 (25 %), median or Q2 (50 %), and upper quartile or Q3 (75 %), respectively. Other examples for quantiles are “percentiles,” which divide the distribution into 100 blocks (i.e., each block comprises a proportion of 1 %), or “deciles,” which divide the distribution into 10 blocks of each 10 % value distribution (Sheskin, 2003). While only three tools explicitly support quartiles, we discovered the availability of other types of quantiles too (in our survey rated as *p*). Table 6 shows the results for the DP-9 test case where quartiles or other types of quantiles are calculated for the column OrderItem.UnitPrice.

**Table 6:** Data profiling—test case quartiles.

	**Q1 (25 %)**	**Q2 (50 %)**	**Q3 (75 %)**	**Type of Quantile**
Aggregate Profiler	12	18.4	32	Quartiles (4 blocks)
DataCleaner	12	18.4	32	Quartiles (4 blocks)
Talend Open Studio	12	18.4	32	Quartiles (4 blocks)
SAS Data Quality	12.9375	19.475	33.4375	Demi-deciles (20 blocks)
InfoZoom & IZDQ	12 (25.48 %)	18.4 (50.63 %)	32 (75.36 %)	Inverse function (2.155 blocks)
Ataccama ONE	0 %: 2, 10 %: 7.45, 20 %: 10, 30 %: 13.25, 40 %: 16, 50 %: 18.4, 60 %: 21.5, 70 %: 30, 80 %: 35.1, 90 %: 46, 100 %: 263.5			Deciles (10 blocks)

Ataccama ONE supports deciles, which are displayed extra in the last row since they cannot be directly mapped to quartiles. Although SAS Data Quality provides 20 blocks, that is, demi-deciles, the functionality is described in the SAS UI as “percentiles,” which would refer to the 100-partitions quantiles. In Table 6, we picked only the values for the quartile blocks out of the 20 blocks in total. In InfoZoom, the inverse function to quantiles is chosen: instead of merging values into blocks, the percentage value of the distribution is display for each value [denoted as “Cumulative Distribution Function” (Dasu and Johnson, 2003)], leading to a total of 2.155 blocks, where each block contains exactly one value. Table 6 displays the percentage value of the distribution that refers to Q1, Q2, and Q3, respectively. It can be summarized that the determination of quantiles is interpreted differently in the single DQ tools with respect to the notation (“Q1” vs. “lower quartile” vs. 25 %) as well as the type of quantile.

##### 5.2.1.3. Single Column—Patterns, Data Types, and Domains

In this category, the support of the different requirements varies widely and there is definitive potential for improvement with respect to out-of-the-box pattern and domain discovery. Even the discovery of basic types (DP-11) is not always supported. For example, DataCleaner recognizes the difference between string, boolean, and number and uses this information for further internal processing, but does not explicitly display it per attribute. While the test cases for the DBMS-specific data types (DP-12) yielded uniform results (“varchar” for ProductName, “decimal” for UnitPrice, and “bit” for isDiscontinued), the variety in terminology and classification for the basic types is outlined in Table 7. In SAS, we had problems to access a table containing the “decimal” data type and thus, converted Product.UnitPrice to “long.” “Alphanumeric” in Experian Pandora is abbreviated with “Alphanum.”

**Table 7:** Data profiling—test cases basic types.

	**A-ONE**	**DM**	**EP**	**IDQ**	**IZDQ**	**OR**	**O-EDQ**	**SAS**
ProductName	String	Text	Alpha- numeric	String(32)	String	String	Text	String
UnitPrice	String	Number	Decimal	Decimal(5,2)	####.##	String/ numeric	Numeric	Long
isDiscontinued	Integer	Number	Integer	Integer(1)	####	Numeric	Numeric	Bit

For the measurement of the value length (DP-13), the minimum (min.) and maximum (max.) values are usually provided, but not always an average (avg.) value length. The median (med.) value length is only provided by Ataccama ONE. We rated this requirement as fulfilled if at least the minimum, maximum, and average value length were provided, considering the median as optional. Table 8 shows the exact results delivered by the single tools, which justifies the fulfillment ratings and indicates differences in the accuracy of the average values. InfoZoom provides only the maximum value length, while SAS and Talend OS restrict this feature to string values.

**Table 8:** Data profiling—test case value length.

	**AP**	**A-ONE**	**DC**	**EP**	**IDQ**	**IZDQ**	**OR**	**O-EDQ**	**SAS**	**TOS**
ProductName (min.)	4	4	4	4	4	−	4	4	4	4
ProductName (max.)	32	32	32	32	32	32	32	32	32	32
ProductName (avg.)	16.269	16.27	16.269	16.32	−	−	16.269	−	−	16.32
ProductName (med.)	−	15	−	−	−	−	−	−	−	−

For the number of digits and decimals, the DQ tools usually use the values documented by the DBMS, e.g., 12 digits and 2 decimals for attribute UnitPrice in table Product, compared to maximum 5 digits and 2 decimals in the real data. Value patterns and their visualization as a histogram (DP-16) is supported by most DQ tools. SAS supports pie charts only.

Generic semantic data types (DP-17), such as code, indicators, date/time, quantity, or identifier are also denoted as “data class” (Abedjan et al., 2015) and are defined by generic patterns. A semantic domain (DP-18), “such as a credit card, first name, city, [or] phenotype” (Abedjan et al., 2015), is more concrete than a generic semantic data type and usually associated with a specific application context. The DQ tools that fulfill these requirements offer a number of patterns, which are associated with the respective generic data type or semantic domain. By applying these patterns to the data values, it could be verified to which extent an attribute contains values that are of a specific type. Thus, the two requirements DP-17 and DP-18 are usually not distinguished within the DQ tools we evaluated. The number of available patterns varies between approximately 10–50 patterns (Pandora, DataCleaner, SAS), 50–100 patterns (Talend), and 100–300 (Informatica, Oracle). While most tools display the matching patterns per attribute (e.g., Product.UnitPrice conforms to 98.72 % to the domain “Geocode_Longitude” using Informatica DQ), SAS displays the matching attribute per pattern (e.g., “Country” matches to 100 % the attribute Customer.Country). Talend OS is the only tool that displays the matching rows instead of the percentage of matching rows per attribute. For Ataccama ONE, we rated DP-17 and DP-18 as partially fulfilled, since specific attributes are classified (e.g., Customer.FirstName as “first name”), but those terms are part of the Ataccama business glossary, which we were unable to access during our evaluation and, therefore, had no further information about its origin.

##### 5.2.1.4. Dependencies

The dependency section has the lowest coverage of the data profiling category and is best supported by Experian Pandora and Informatica DQ (in Developer edition only). Although we introduce each concept briefly, we refer to Abedjan et al. (2019) for details about dependency discovery and their implementation. In the following, R denotes a relational schema (defining a set of attributes) with *r* being an instance of R (defining a set of records). Sets of attributes are denoted by α and β.

A unique column combination (UCC) is an attribute set α⊆R whose projection contains no duplicate entries in *r* (Abedjan et al., 2019). In other words, a UCC is a (possibly composite) candidate key that functionally determines R. While Experian Pandora allows the detection of single column keys (thus *p*), Informatica DQ offers full UCC detection. Both tools allow the user to set a threshold for relaxed UCC detection (DP-20) and to identify violating records *via* drill-down. With Informatica DQ, we discovered five UCCs in table Order of our test DB, using a threshold of 98 %: Id (100 %), OrderNumber (100 %), OrderDate + TotalAmount (100 %), CustomerId + TotalAmount (99.88 %), and CustomerId + OrderDate (99.16 %). With Experian Pandora, only the two single column keys Id (100 %) and OrderNumber (100 %) were detected. SAS Data Quality indicates 100 % unique attributes as primary key candidates.

An inclusion dependency (IND) over the relational schemata *R*_*i*_ and *R*_*j*_ states that all values in attribute set α also occur in β, that is *R*_*i*_[α]⊆*R*_*i*_[β] (Abedjan et al., 2019). The detection of INDs (DP-21 and DP-22), also referred to as foreign key discovery, is not widely supported. The best automation for this requirement delivers Experian Pandora, where initially the primary keys (UCCs) and foreign key relations are inferred, and based on this information, INDs are displayed graphically as a Venn diagram. In addition, it is possible to drill down to records that violate those INDs in a spread sheet. Informatica DQ and SAS Data Quality support IND discovery only partially, since it is required that the user selects the respective primary key (UCCs) and assigns it to possible foreign key candidates that are then tested for compliance. DataCleaner can only be used to check if two tables can possibly be joined, without information on the respective columns or the join quality (i.e., violating rows).

A functional dependency (FD) α → β asserts that all pairs of records with the same attribute values in α must also have the same attribute values in β. Thus, the α-values functionally determine the β-values (Codd, 1970). Again, we used table Order to verify exact (DP-23) and relaxed (DP-24) FD detection. With Experian Pandora and Informatica DQ we found in total eight exact FDs: {Id} → {OrderDate, OrderNumber, CustomerId, TotalAmount} and {OrderNumber} → {Id, OrderDate, CustomerId, TotalAmount} and two more FDs when relaxing the threshold to 93 %: {TotalAmount} → {CustomerId, OrderDate}. Talend OS fulfills FD discovery only partially, because it requires user interaction to specify the attribute sets α and β, given that the number and type of columns are equal. Although specific FDs can be tested with this functionality (e.g., to which extent TotalAmount determines CustomerId), we do not perceive it as true automated FD discovery, e.g., when performing the test case and specifying all attributes of Order as α and β respectively, the result are five FDs, where each attribute is discovered to functionally determine itself. This case should be ideally excluded during the detection. All three tools printed the identified FDs in table-format, one row for each attribute pair along with the match percentage, but with slightly differing terminology. Thus α (the left side) is denoted as “A column set,” “identity column” or “determinant column,” and β (the right side) is denoted as “B column set,” “identified columns” or “dependent column.” For relaxed FDs, Experian displayed the violating rows with a count (50 in this case), Informatica listed the respective rows, and Talend did not provide violating rows at all.

##### 5.2.1.5. Advanced Multi-Column Profiling

Apart from duplicate detection, which is a widely supported feature, advanced multi-column features are rarely supported satisfactorily. No single tool offers association rule mining (DP-26) as mentioned by Abedjan et al. (2015). Note that we specifically tested the DQ tools described in Section 5.1, and did not consider related tools that are often installed together. For example, SAS Enterprise Guide, which was shipped with our DQ installation, is dedicated to data analysis and therefore provides a rich function palette that overlaps with the multi-column profiling section, e.g., a selection of correlation coefficients, hierarchical and k-means clustering. Since the aim of this survey is to investigate DQ tools, we did not consider such related tools.

Correlations (DP-25) are a statistical measure between 1.0 and -1.0 to indicate the relationship between two numerical attributes (Sheskin, 2003; Abedjan et al., 2015). The most commonly used coefficients are Pearson correlation coefficient, or the rank-based Spearman's or Kendall's tau correlation coefficients (Sheskin, 2003). In our survey, only Aggregate Profiler is able to compute Pearson correlations. However, our test case for DP-25 (Pearson correlation between OrderItem.UnitPrice and OrderItem.Quantity) yielded -0.045350608 with Aggregate Profiler, which did not conform to our cross-check using SAS Enterprise Guide (0.00737) and the Python package numpy (0.00736647). Talend distinguishes between “numerical,” “time,” and “nominal” correlation analysis and displays the respective correlations in bubble charts. We rated this as partial fulfillment *p*, since no correlation coefficient is calculated and the calculation is restricted to single columns with specific data types, thus, it is not possible to calculate the correlation between two interval data types.

During our investigation, we found that the concepts of clustering (DP-27), outlier detection (DP-28), and duplicate detection (DP-29 and DP-30) are not always clearly distinguishable in practice. Also Abedjan et al. (2015) state that clustering can be either used to detect outliers in a single column, or to detect similar or duplicate records within a table. Thus, we describe the three concepts briefly along with the condition we applied to verify (partial) fulfillment of the respective requirement.

Clustering (DP-27) is a type of unsupervised machine learning, where data items (e.g., records or attribute values) are classified into groups (clusters) (Jain et al., 2000). A comprehensive overview on existing clustering algorithms is provided by Jain et al. (2000). In some DQ tools, clustering is only available in the frame of duplicate detection. For example, in OpenRefine clustering is used to detect duplicate string values (cf. Stephens, 2018), in Informatica DQ the grouped duplicates are referred to as “clusters,” SAS Data Quality requires a “clustering” component to group records based on their match codes (SAS, 2019); and Oracle EDQ uses clustering as preprocessing step of the matching component to increase runtime efficiency by preventing unnecessary comparisons between records (Oracle, 2018). To completely fulfill requirement DP-27, we presumed the availability of one of the common clustering algorithms (like *k*-means or hierarchical clustering) as an independent function. Datamartist supports *k*-means clustering and allows to select the number of clusters k from 5 predefined values (5, 10, 25, 50, 100) and to restrict the observed value range. Aggregate Profiler supports *k*-means clustering without any modification possibility (e.g., choose *k*), as well as a second type of clustering for numeric values, where the number of clusters can be defined. No further information about this clustering algorithm is provided. No tool offers hierarchical clustering or other partitional clustering algorithms except *k*-means, for example, graph theoretic approaches or expectation maximization (Jain et al., 2000).

Outlier detection deals with data points that are considered abnormalities, deviants, or discordant when compared to the remaining data (Aggarwal, 2017). A comprehensive overview on different algorithms to detect outliers is provided by Aggarwal (2017). Our investigation showed that outlier detection is implemented in the tools very differently, and compared to the current state of research, only simple methods are used. We have not found a tool that supports multivariate outlier detection or one of the more sophisticated approaches like z-score, linear regression models, or probabilistic models as mentioned by Aggarwal (2017). In the following, we describe the implementation of outlier detection and the result that our test case yielded to “find very high values” in Order.TotalAmount:

- Aggregate Profiler, Ataccama ONE, Datamartist, and InfoZoom provide outlier detection for numerical values only visually, either in a quantile plot (Ataccama), in a bar chart (Datamartist) or in form of a box plot. Aggregate Profiler and Ataccama ONE do not allow drill-down to the actual outlying values and in InfoZoom the visualization of the single values in the plot are not readable. In all three tools, it is not possible to modify the plot settings or to get details about the used settings. The bar chart in Datamartist is based on k-means clustering with the same modification options as described in the previous paragraph. When using the standard settings (100 bars), one outlier is detected for our test case of finding “very high values” in column Product.UnitPrices: 17250.0. This extreme value is detected by all tools correctly, although other methods yield more outlying values.
- Experian Pandora offers a number of different types of outlier checks, where some require one of the two parameters that can be specified by a user: “Rarity Threshold” (default: 1000) and “Standard Deviation Tolerance” (default: 3.3) (Experian, 2018). The rarity threshold is used to detect rare values, which occur less frequently than one time in < threshold> is used for the checks “rare values,” “is a key,” and “unusually missing values” (Experian, 2018). The standard deviation tolerance specifies the number of standard deviations that is tolerated for a value to be apart from the norm. It is used for low/high amounts, short/long values, rare/frequent values, and rare formats (Experian, 2018). By using the standard settings, we found 18 outlying values for our test case (17250.0, 16321.9, 15810.0, 12281.2, 11493.2, 11490.7, 11380.0, 11283.2, 10835.24, 10741.6, 10588.5, 10495.6, 10191.7, 10164.8, 8902.5, 8891.0, 8623.45, 8267.4).
- Informatica DQ distinguishes between “pattern outliers,” which refer to unusual patterns in the data, and “value frequency outliers” (Informatica, 2018), where values with unusual occurring frequency are displayed. With this functionality, it was not possible to perform our test case, because the characteristic of being an outlier depends on the frequency instead of the actual value.
- SAS provides an “outliers” tab for columns of different data types, where a fixed number of five minimum and maximum values are outlined without any modification possibility. For our test case, the following maximum values have been detected: 17250.0, 16321.0, 15810.0, 12281.2, and 11493.2, which correspond to the five highest results detected with Pandora.

Duplicate detection “aims to identify records [...] that refer to the same real-world entity” (Elmagarmid et al., 2006). It is a widely researched field, which is also referred to as record matching, record linkage, data merging, or redundancy detection (Elmagarmid et al., 2006). In contrast to clustering and outlier detection, the understanding and implementation of duplicate detection is very similar across all tools we investigated. In principle, the user (1) selects the columns that should be considered for comparison, (2) optionally applies a transformation to those columns (e.g., pruning a string to the first three characters), and finally (3) selects an appropriate distance function and algorithm. The major difference in the implementations is the selection of distance functions for the attribute values. The following distances are supported:

- Aggregate Profiler: exact match, similar-any word (if any word is similar for this column), similar-all words (if all words are similar for this column), begin char match, and end char match (Arrah Technology, 2019). No information about the used similarity function was provided.
- DataCleaner: n-grams, first 5, last 5, sorted acronym, Metaphone, common integer, Fingerprint, near integer (for pre-selection phase); exact, is empty, normalized affine gap, and cosine similarity (for scoring phase). The two phases are explained in the following paragraph.
- Experian Pandora: Edit distance, exact, exact (ignore cases), Jaro distance, Jaro-Winkler distance, regular expression, and Soundex.
- Informatica DQ: Bigram, Edit, Hamming, reverse Hamming, and Jaro distance.
- InfoZoom: Soundex and Cologne phonetics.
- OpenRefine: Fingerprint, n-gram Fingerprint, Metaphone3, or Cologne phonetics (with key collision method); Levenshtein or PPM (with nearest neighbor method) cf. (Stephens, 2018) for details.
- Oracle EDQ: (transformations) absolute value, first/last n characters/words, lower case, Metaphone, normalize whitespace, round, Soundex.
- Talend OS: exact, exact (ignore case), Soundex, Soundex FR, Levenshtein, Metaphone, Double Metaphone, Jaro, Fingerprint key, Jaro-Winkler, q-grams, Hamming, and custom.

SAS Data Quality does not offer string distances, but matches based on match codes (SAS, 2019), which are generated based on an input variable, a “definition” (type of transformation for the input variable) and a “sensitivy” (threshold), where records with the same match codes are then grouped together into the same cluster. The list of match definitions depends on the used Quality Knowledge Base (QBK). Talend OS offers two different algorithms to define the record merge strategy: simple VSR Matcher (default) or T-Swoosh. We refer to the documentation (Talend, 2017) for details.

DataCleaner implements a ML-based approach that distinguishes between two modes: untrained detection (considered experimental) and a training mode plus duplicate detection using the trained ML model (Quadient, 2008). The training mode is divided into three phases: (1) pre-selection, (2) scoring using a random forest classifier and the distance functions mentioned above, and (3) the outcome, which highlights duplicate pairs with a probability between 0 and 1.

Despite the fact that duplicate detection is typically attributed toward data cleansing (data integration or data matching) and not considered to be part of data profiling in the implementations, most DQ tools allow this functionality to be used also for detection purposes. We rated Datamartist and Aggregate Profiler as supporting this requirement partially since the function is dedicated to direct cleansing (deletion or replacement of records) and because of the very limited configuration options compared to all other tools. Datamartist does not support DP-30 at all.

#### 5.2.2. Data Quality Measurement Capabilities

Table 9 summarizes the fulfillment of the DQM category, where the first part is dedicated to DQ dimensions, and the second one to business rules.

**Table 9:** Data quality measurement capabilities.

		**Aggregate Profiler**	**Apache Griffin**	**Ataccama ONE**	**DataCleaner**	**Datamartist**	**Experian Pandora**	**Informatica DQ**	**InfoZoom & IZDQ**	**MobyDQ**	**OpenRefine & MetricDoc**	**Oracle EDQ**	**SAS Data Quality**	**Talend Open Studio**
31	Accuracy metric	−	✓	−	−	−	−	−	−	−	−	−	−	−
32	Completeness metric	−	−	*p*	*p*	*p*	*p*	*p*	*p*	✓	−	−	−	−
33	Consistency metric	−	−	−	−	−	−	−	−	−	−	−	−	−
34	Timeliness metric	−	−	−	−	−	−	−	−	−	−	−	−	−
35	Other DQ metrics	−	−	−	*p*	*p*	*p*	*p*	−	✓	✓	−	*p*	*p*
36	Creation of business rules	✓	−	−	−	✓	✓	✓	✓	✓	✓	✓	✓	✓
37	General-applicable rules	−	−	−	−	−	✓	✓	*p*	−	−	−	✓	✓
38	Application of business rules	✓	−	−	−	✓	✓	✓	✓	✓	✓	✓	✓	✓

##### 5.2.2.1. Accuracy

An accuracy metric (on table-level) is only provided by Apache Griffin, where the user needs to select a source and a target table and accuracy is calculated according to $A_{tab}=\frac{\left|r_{a}\right|}{\left|r\right|}*100\%$, where |*r*| is the total number of records in the source table, and |*r*_*a*_| the number of (accurate) records in the target table that can be directly matched to a record in the source table (Apache Foundation, 2019). This metric corresponds to the accuracy metric proposed by Redman (2005), which is outlined in Equation (2).

##### 5.2.2.2. Completeness

The metric for the completeness on attribute-level ($C_{att}=\frac{\left|v_{c}\right|}{\left|v\right|}$) introduced in Equation (8) is closely related to DP-3, the percentage of null values, which yields the missingness $M_{att}=\frac{\left|v_{n}\right|}{\left|v\right|}=1-C_{att}$, where |*v*_*n*_| is the number of null values in one column. Ataccama, DataCleaner, Datamartist, Experian, and InfoZoom provide the completeness calculation on attribute-level according to Equation (8), without the possibility for aggregation on higher levels. Informatica DQ allows an aggregation on table-level (as the arithmetic mean of all attribute-level completeness values), but not higher. Note that this aggregated metric differs from the table-level completeness proposed by (Hinrichs, 2002) and described in Equation (7), which calculates the mean of all completeness values at the record-level. Despite the fact that MetricDoc offers a metric that is denoted “completeness” in the GUI and is also described in their scientific documentation (Bors et al., 2018), they calculate the missingness *M*_*att*_ on attribute-level and thus did not fulfill requirement DQM-32. MobyDQ computes the completeness between two data sources (source and target) according to $C_{tab}=\frac{C_{t}-C_{s}}{C_{s}}$, where *C*_*t*_ is the completeness measure of the target source and *C*_*s*_ the measure from the source. If *C*_*s*_ is considered to be the reference dataset, this metric corresponds to the completeness calculation proposed by Batini and Scannapieco (2016), which is discussed in Section 2.2.2.

##### 5.2.2.3. Consistency

The consistency dimension is mentioned in the Informatica DQ methodology (Informatica, 2010), and SAS implements no single metric, but a set of rules that are grouped to this dimension, e.g., checks if an attribute contains numbers, non-numbers, is alphabetic, or is all lower case. We did not rate this as fulfilled because no aggregate metric was provided to calculate “consistency” and these rules are supplied by most DQ tools with generally applicable business rules (DQM-37). However, we would like to point out that the understanding of Informatica and SAS corresponds to the consistency metrics proposed in research (cf. Section 2.2.3), since both approaches are rule-based. In contrast to a predefined metric, Informatica and SAS assume that the user creates metric manually.

##### 5.2.2.4. Timeliness

We did not find an implementation for the timeliness dimension as discussed in Section 2.2.4. However, with respect to other time-related dimensions, MetricDoc offers two different *time interval metrics*, where one checks if the interval between two timestamps “is smaller than, larger than, or equal to a given duration value” (Bors et al., 2018), and the second one performs outlier detection on interval length. MobyDQ offers metrics for *freshness* and *latency*, but refers to those dimensions as DQ “indicators” (Rolland, 2019). Freshness is implemented as *ts*_*cur*_ − *ts*_*t*_, where *ts*_*cur*_ is the current timestamp and *ts*_*t*_ the last updated timestamp from the target request, and latency as *ts*_*s*_ − *ts*_*t*_, where *ts*_*s*_ is the last updated timestamp from the source request (Rolland, 2019). These indicators are not specifically dedicated to DQ dimensions and do not fulfill the requirement for DQ metrics to be normalized between [0,1] by Heinrich et al. (2018).

##### 5.2.2.5. Other DQ Metrics

With respect to other non-time-related DQ metrics (DQM-35), the *uniqueness* dimension is most often implemented according to $U_{att}=\frac{\left|v_{u}\right|}{\left|v\right|}$, where |*v*_*u*_| refers to the number of unique values within a column. DataCleaner, Datamartist, Experian, SAS Data Quality, and Talend OS implement uniqueness on attribute-level only, which corresponds to the requirement DP-5. Informatica DQ allows an aggregation on table-level but not higher. MetricDoc implements a dimension referred to as “uniqueness,” but actually calculate the *redundancy* $R_{tab}=\frac{\left|r_{black}\right|}{\left|r\right|}$ on table-level, where |*r*_*black*_| is the number of records with at least one duplicate entry in the table. The user needs to select more than one attribute within a table in order to calculate the metric and it cannot be aggregated on higher levels. In addition, MetricDoc offers metrics for the DQ dimensions *validity* and *plausibility*. Validity is calculated on attribute-level as $V_{att}=\frac{\left|v_{i}\right|}{\left|v\right|}$, where |*v*_*i*_| is the number of attribute values that do not comply to the column data type. Plausibility is also calculated on attribute-level as $P_{att}=\frac{\left|v_{j}\right|}{\left|v\right|}$, where |*v*_*j*_| is the number of attribute values that are outliers according to a nonrubost or robust statistical measure (mean with standard deviation, or median with interquartile range estimator, respectively) (Bors et al., 2018). MobyDQ also provides a *validity* indicator, which connects to one single target data source and compares the values with defined thresholds (Rolland, 2019). SAS does not provide predefined metrics, but uses DQ dimensions as abstraction layer to group business rules.

A number of additional DQ dimensions are mentioned in documentations or on websites of DQ tool vendors without being implemented as metric. In order to provide a structured overview, these additionally mentioned DQ dimensions are summarized together with all “other” DQ dimensions and their implementations (explained in this paragraph) in the following list:

- *Conformance*: mentioned by Informatica Loshin (2006).
- *Conformity*: mentioned by Informatica (2010).
- *Correctness*: mentioned in DataCleaner documentation (Quadient, 2008).
- *Currency*: mentioned by Loshin (2006).
- *Duplicates*: mentioned by Informatica (Informatica, 2010).
- *Duplication*: mentioned in DataCleaner documentation (Quadient, 2008).
- *Freshness*: implemented by MobyDQ as *ts*_*cur*_ − *ts*_*t*_ (Rolland, 2019).
- *Integrity*: mentioned in Informatica (2010), Talend (2017), and SAS (2019).
- *Latency*: implemented by MobyDQ as *ts*_*s*_ − *ts*_*t*_ (Rolland, 2019).
- *Plausibility*: implemented by MetricDoc for OpenRefine as $P_{att}=\frac{\left|v_{j}\right|}{\left|v\right|}$ (Bors et al., 2018).
- *Referential Integrity*: mentioned by Informatica (Loshin, 2006).
- *Structure*: mentioned by SAS (2019).
- *Uniformedness*: mentioned in DataCleaner documentation (Quadient, 2008).
- *Uniqueness*: implemented by DataCleaner, Datamartist, Experian, SAS Data Quality, and Talend OS on attribute-level as $U_{att}=\frac{\left|v_{u}\right|}{\left|v\right|}$ and by Informatica also on table-level. Implemented by MetricDoc for OpenRefine as $R_{tab}=\frac{\left|r_{black}\right|}{\left|r\right|}$. Also mentioned in Loshin (2006); Datamartist (2017); SAS (2019); Experian (2020).
- *Validity*: implemented by MetricDoc for OpenRefine as $V_{att}=\frac{\left|v_{i}\right|}{\left|v\right|}$ (Bors et al., 2018). Also mentioned in SAS (2019) and Experian (2020).

##### 5.2.2.6. Business Rules

While the creation and application of business rules (DQM-36 and DQM-38) is supported by most DQ tools, few tools also offer predefined generally applicable business rules. A widely supported example are rules for address validation (e.g., zip codes, cities, states) that tackle the prevalent problem of failed mail deliveries due to incorrect addresses, also described by Apel et al. (2015). Despite the good performance of DataCleaner in terms of data profiling and CDQM, it does not support business rules at all. We rated DP-37 (the availability of generally applicable rules) as only partly fulfilled for InfoZoom, because the provided rules have been created for a given demo DB schema and would need to be modified to apply to other schemas (e.g., with other column names).

#### 5.2.3. Data Quality Monitoring Capabilities

The results of the CDQM evaluation are shown in Table 10. We want to point out that for two DQ tools (Ataccama ONE and Talend OS), more advanced versions are available that support CDQM according to their vendors, but we did not investigate it.

**Table 10:** Data quality monitoring capabilities.

		**Aggregate Profiler**	**Apache Griffin**	**Ataccama ONE**	**DataCleaner**	**Datamartist**	**Experian Pandora**	**Informatica DQ**	**InfoZoom & IZDQ**	**MobyDQ**	**OpenRefine & MetricDoc**	**Oracle EDQ**	**SAS Data Quality**	**Talend Open Studio**
39	Task scheduling	*p*	✓	−	✓	*p*	✓	✓	*p*	✓	−	✓	*p*	−
40	Storage of results	✓	✓	✓	✓	✓	✓	✓	✓	✓	✓	✓	✓	✓
41	Retrieval of results	−	✓	✓	✓	−	−	✓	✓	−	−	✓	✓	✓
42	Comparison	−	✓	−	✓	−	✓	✓	✓	−	−	−	*p*	−
43	Visualization over time	−	✓	−	✓	−	✓	✓	✓	−	−	−	*p*	−

The storage (CDQM-40) of DP or DQM results is possible in all tools. The majority of DQ tools (except MobyDQ and OpenRefine) also support data export *via* a GUI. Datamartist allows to export only very basic data profiles. The most comprehensive enterprise solutions for CDQM-40 and 41 is provided by Informatica DQ and SAS Data Quality, which enable the export of full DP procedures. During import, all settings and required data sources are reloaded from the time of the analysis.

Task scheduling (CDQM-39) is also widely supported. Aggregate Profiler fulfills this requirement only partially, since it is only possible to schedule business rules, but no other form of tasks, e.g., data profiling tasks. With Datamartist, InfoZoom, and SAS Data Quality, task scheduling is cumbersome for business users, since the command line is required to write batch files. With Datamartist, the tool needs to be closed to execute the batch file.

To visualize the continuously performed DQ checks (be it DP tasks, user-defined rules, or DQ metrics), Informatica DQ relies on so called “scorecards,” which can be customized to display the respective information. Apache Griffin, Experian Pandora, and SAS Data Quality also allow alerts to be defined, when specific errors occur or when a defined rule is violated. MobyDQ does not offer any visualization (which is considered future work), but relies on external libraries in its implementation at Ubisoft. SAS fulfills both requirements CDQM-42 and 43 only partially, since its “dashboards” contain solely the number or percentage of triggers per date, source or user, but no specific values (e.g., 80 % completeness) could be plotted. The most comprehensive solution for CDQM in general-purpose DQ tools provide Informatica DQ and DataCleaner by Human Inference. With respect to the open-source tools, only Apache Griffin provides comprehensive CDQM support, and the commercial version of MobyDQ, which is deployed at Ubisoft.

## 6. Survey Discussion and Lessons Learned

The results of our survey on DQ measurement and monitoring tools revealed interesting characteristics of DQ tools and allow to draw conclusions about the future direction of automated and continuous data quality measurement. While the following paragraphs provide a general overview on the marked of DQ tools, which was a side-result of this survey, each sub-question and the overall research question of this survey are discussed separately per subsection.

One of the greatest challenges we faced during the conduct of this survey was the constant change and development of the DQ tools, especially the open-source tools. Nevertheless, it is of great value to reflect on the current state of the market for two reasons: (1) to create a uniform vision for the future of DQ research, and (2) to identify the potential for functional enhancement across the tools.

The fact that we found 667 tools attributed to “data quality” in our systematic search indicates the growing awareness of the topic. However, approximately half (50.82 %) of the DQ tools that we found were domain specific, which means they were either dedicated to specific types of data or built to measure the DQ of a proprietary tool. This amount underlines the “fitness for use” principle of DQ, which states that the quality of data is dictated by the user and type of usage. 40 % of the DQ tools were dedicated to a specific data management task, for example, data cleansing, data integration, or data visualization, which reflects the complexity of the topic “data quality.” Although those tasks are often not clearly distinguished in practice, we required explicit DQ measurement, that is, making statements about the DQ without modifying the observed data.

Our selection of DQ tools provides a good digest of the market, since we included eight commercial and closed-source tools as well as five free and open-source tools, from which four (except Talend) are not mentioned by Gartner (cf. Chien and Jain, 2019). The vendors of four tools have been named “leader” in the Magic Quadrant of Data Quality Tools 2019 (Informatica, SAS, Talend, Oracle) and two of them are among the four vendors currently controlling the market [which are SAP, Informatica, Experian, and Syncsort (Chien and Jain, 2019); however, no trial for SAP nor Syncsort was granted].

Overall and according to our requirements, we experienced Informatica DQ as the most mature DQ tool. The best support for data profiling is provided by Experian Pandora, which allows to profile across an entire DB and even across multiple connected data sources. All other tools allow data profiling only for selected columns or within specific tables. Despite being classified as leader by Gartner, we perceived Oracle EDQ, Talend OS, and SAS Data Quality as having less support for data profiling and/or DQ monitoring. Although Quadient (with DataCleaner) was removed from the Gartner study in 2019 due to their focus on customer data, our evaluation yielded a good support in data profiling and a strong support in DQ monitoring. However, when comparing the two general-purpose and freely available DQ tools Talend OS and Aggregate Profiler, the former one convinced in terms of intuitive user interface and a good overall performance. Aggregate Profiler on the other hand, has a richer support for advanced multi-column profiling and data cleansing, but it is not always clear which algorithms are used to perform data modifications and the documentation is not up-to-date.

Three open-source tools (Apache Griffin, MobyDQ, and OpenRefine) were installed from GitHub and thus required technical knowledge for the setup. While OpenRefine can not keep up with comparable tools like Talend OS or Aggregate Profiler in terms of data profiling, MobyDQ and Apache Griffin have clearly a different focus on CDQM. IBM ISDQ demonstrated, that also commercial tools can be very arduous and time intensive to install due to the increasing complexity of the single modules and dependencies between them.

### 6.1. Data Profiling Capabilities in Current DQ Tools

In order to answer the first sub-research-question “*Which data profiling capabilities are supported by current DQ tools?*” we compiled 30 requirements that are mainly based on the classification on data profiling by Abedjan et al. (2019).

In summary, 11 (all except Apache Griffin and MobyDQ) of the 13 tools examined supported data profiling at least partially. The details on the data profiling capabilities per DQ tools are discussed in Section 5.2.1. Our evaluation revealed that especially single-column data profiling like cardinalities (DP 1—5) were supported by all 11 tools. However, considering the state-of-the-art in research, there is potential for functional enhancement with respect to multi-column profiling (DP 25–30) and dependency discovery (DP 19–24). For example, dependency discovery is only supported by 2 tools in a comprehensive way. While in the group of multi-column profiling, exact and approximate duplicate detection is a very common feature (supported at least partially by 10 tools in total), correlation analysis is only supported by one tool (Aggregation Profiler) completely, and a second tool (Talend Open Studio) partially. Association-rule mining is not supported by any tool at all and there is also no full support for clustering by any tool observed. According to our customer contacts and reference customers, those functionalities are not considered to be part of data profiling and are usually implemented in analytics tools (e.g., SAS Enterprise Guide). This might be a reason why most DQ tools in our evaluation lack a wide range of features in this category: customers and vendors simply do not consider it as part of data profiling and data quality. This observation can be explained by the unclear distinction between the terms “data profiling” and “data mining.” Abedjan et al. (2019) distinguish the two topics by the object of analysis (focus on *columns* in data profiling vs. *rows* in data mining) and by the goal of the task (gathering technical metadata by data profiling vs. gathering new insights by data mining) (Abedjan et al., 2019). While this distinction is still fuzzy, we go one step further and claim that there is also no clear distinction between data mining and data analytics with respect to the used techniques [e.g., regression analysis is discussed in both topics (Dasu and Johnson, 2003)].

In recent years, numerous research initiatives concerning data profiling have been carried out that also use ML-based methods. Current general-purpose DQ tools do not take full advantage of these features. Although, several vendors claim to implement ML-based methods, we found no or only limited documentation of concrete algorithms (cf. Quadient, 2008). Note that in the case of DataCleaner for duplicate detection, we received more detailed documentation upon request. We think that especially concerning the hype for artificial intelligence and the enhancement of detecting DQ errors with ML methods, it is necessary to focus on the desirable core characteristics for DQ and data mining (Dasu and Johnson, 2003): the methods should be widely applicable, easy to use, interpret, store and deploy, and should have short response times. A counterexample are neural networks, which are increasingly applied in recent research initiatives, but need to be handled with care for DQ measurement, because they are black-box and hard to interpret. For measuring the quality of data (to ensure reliable and trustworthy data analysis), easy and clearly interpretable statistics and algorithms are required to prevent a user from deriving wrong conclusions from the results.

Apart from functional enhancements, we want to point out the desire for more automation and in data profiling. Current DQ tools allow users to select data profiling features or to define rules, which are then applied to single attributes or tables. This does not meet today's requirements to master big data problems, where typically, multiple information systems needs to be monitored at the same time (Stonebraker and Ilyas, 2018). To ease the high initialization effort for large information system infrastructures, more automated, initial and still meaningful out-of-the-box profiling would be required.

### 6.2. Data Quality Measurement Capabilities

The first sub-research-question “*Which data quality dimensions and metrics can be measured with current DQ tools?*” was inspired by the number of DQ dimensions and metrics proposed by researchers (cf. Piro, 2014; Batini and Scannapieco, 2016; Heinrich et al., 2018 and the detailed outline in Section 2.2). In our survey, did not find a tool that implements a wider range of DQ metrics for the most important DQ dimensions as proposed in research papers and we also did not find another survey that investigates the existence of DQ metrics in tools. Identified DQ metric implementations have several drawbacks: some are only applicable on attribute-level (e.g., no aggregation possibility), some require a gold standard that might not exist, and some have implementation errors.

#### 6.2.1. DQ Metrics for Accuracy and the Problem of Gold Standards

The two open-source tools that implement metrics for the DQ dimensions accuracy (Apache Griffin) and completeness between two tables (MobyDQ) relied on a reference data set (i.e., gold standard) provided by the user. Apache Griffin based their metric on the definition by DAMA UK, who state that accuracy is “the degree to which data correctly describes the ‘real world' object or event being described” (Askham et al., 2013), which needs to be selected for the calculation. MobyDQ specifically aims at automating DQ checks in data pipelines, that is, computing the difference between a source and a target data source, where the gold standard is clearly defined. However, in scenarios where the quality of a single data source should be assessed, such metrics are not suitable since a reference or gold standard is often not available (Ehrlinger et al., 2018). This fact is also reflected by the restricted prevalence of such gold-standard-depending DQ metrics in commercial and general-purpose DQ tools.

#### 6.2.2. DQ Metrics for Completeness and Uniqueness

The other investigated tools mainly implement two very basic metrics: completeness (indicating the missing data problem) and uniqueness (indicating duplicate data values or records). It is noteworthy that while completeness is one of the most-widely used DQ dimensions (cf. Batini and Scannapieco, 2016; Myers, 2017; Heinrich et al., 2018), the aspect of uniqueness is often neglected in DQ research (Ehrlinger and Wöß, 2019). For example, (Piro, 2014) perceives duplicate detection as a *symptom* of data quality, but not as DQ dimension. Neither (Myers, 2017) in his “List of Conformed Dimensions of Data Quality,” nor the ISO/IEC 25024:2015 standard on DQ (ISO/IEC 25024:2015(E), 2015) refer to a DQ dimension that describes the aspect of uniqueness or non-redundancy (Ehrlinger and Wöß, 2019). Despite this difference, both DQ dimensions have a common characteristic: they can be calculated without necessarily requiring a gold standard. Nevertheless, these implementations lack two aspects: (1) the aggregation of DQ dimensions and (2) schema-level DQ dimensions that are clearly part of the DQ topic (Batini and Scannapieco, 2016). The aggregation of DQ dimensions from value-level to attribute-, record-, table-, DB- or cross-data-source-level as presented by Hinrichs (2002); Piro (2014) was not provided by any tool prefabricated. Informatica DQ is the only tool that allows to aggregate column-level metrics on table-level, but not higher. We did not declare a manual implementation in tools with strong rule support as availability of such aggregation functions.

#### 6.2.3. DQ Measurement Methodologies

Despite the lack of prefabricated DQ metrics, most tools refer to a set of DQ dimensions in their user guide or defined methodology, for example, Informatica and SAS rely on whitepapers influenced by David Loshin (cf. Informatica, 2010; SAS, 2019), or Talend promotes the existence of such metrics on their website^16^. In Section 5.2.2, we showed that the list of referenced DQ dimensions and metrics by the DQ vendors is very non-uniformly. Further inquiry on the metrics yielded two different responses by our customer contacts: while some explicitly stated that they do not offer generally applicable DQ metrics, others could not answer the question of how specific metrics are implemented.

In the case of Talend, we asked our customer contact and the Talend Community^17^, where the metrics promoted on the website can be found. Unfortunately, we got no satisfying answer, only references to the data profiling perspective in TOS and its documentation. This experience underlines the statement by Sebastian-Coleman (2013) that “people can often not say how to measure completeness or accuracy,” which also leads to different interpretations and implementations.

Other vendors justified the absence of generally applicable DQ metrics with two reasons: because such metrics are not feasible in practice, and because customers do not request it. Several DQ strategies also indicate the fact that DQ metrics should be created by the user and adjusted to the data (cf. Informatica, 2010; Apache Foundation, 2019; SAS, 2019). This understanding follows the “fitness for use” principle, which highlights the subjectivity of DQ. Also Piro (2014) states that objectively measurable DQ dimensions previously require a manual configuration by a user. An example for this is Apache Griffin, who state that “Data scientists/analyst *define* their DQ requirements such as accuracy, completeness, timeliness, and profiling” (Apache Foundation, 2019). Sebastian-Coleman (2013) points out that it is important to understand the DQ dimensions, but these do not immediately lend themselves to enabling specific measurements. The main foundation into DQ measurement, including the set of DQ dimensions and metrics have been originally proposed in the course of the Total Data Quality Management (TDQM) program of MIT^18^ in the 1980s. Dasu and Johnson (2003) state that DQ dimensions, as originally proposed by the TDQM, are not practically implementable and it is often not clear what they mean. The results of our survey underlines this statement with a scientific foundation, because each DQ tool implements the dimensions differently, and partially far away from the complex metrics proposed in research (e.g., no aggregation, often no gold standard). Apart from completeness and uniqueness on attribute-level, no DQ dimension finds wide-spread agreement in the implementation and definition in practice. This is especially noteworthy for the frequently mentioned accuracy dimension, which however, requires a reference data set that is often not available in practice.

#### 6.2.4. The Meaning of DQ Dimensions and Metrics for DQ Measurement

We conclude that there is a strong need to question the current use of DQ dimensions and metrics. Research efforts to measure DQ dimensions directly with a single, generally-applicable DQ metric have little practical relevance and can hardly be found in DQ tools. In practice, DQ dimensions are used to group domain-specific DQ rules (sometimes referred to as metrics) on a higher level. Since research and practitioners failed to create a common understanding of DQ dimensions and their measurement for decades, a complementary and more practice-oriented approach should be developed. Several DQ tools show that DQ measurement is possible without referring to the dimensions at all. Since our focus is the automation of DQ measurement, a practical approach would be required without the need for DQ dimensions, but a focus on the core aspects (like missing data and duplicate detection), which can actually be measured automatically.

### 6.3. Data Quality Monitoring

The third sub-research question addresses “*whether DQ tools enable automated monitoring of data quality over time*.” In contrast to Pushkarev et al. (2010), who did not find any tool that supports DQ monitoring, we identified the existence of this feature, as shown in Table 10.

In general-purpose DQ tools (e.g., DataCleaner, Informatica EDQ, InfoZoom & IZDQ), DQ monitoring is considered a premium feature, which is liable to costs and only provided in professional versions. This is also the reason, why DQ monitoring has not been studied so far in related work that focused on open-source DQ tools (cf. Pushkarev et al., 2010). An exceptions to this observation is the dedicated open-source DQ monitoring tool Apache Griffin, which supports the automation of DQ metrics, but lacks predefined functions and data profiling capabilities. The remaining open question with respect to DQ monitoring is which aspects of the data should actually be measured (discussed in Section 6.2).

## 7. Conclusion and Outlook

In this survey, we conducted a systematic search in which we identified 667 software tools dedicated to the topic “data quality.” With six predefined exclusion criteria, we extracted 17 tools for deeper investigation. We evaluated 13 of the 17 tools with regard to our catalog of 43 requirements divided into the three categories (1) data profiling, (2) DQ measurement, and (3) continuous DQ monitoring. Although the market of DQ tools is continuously changing, this survey gives a comprehensive overview on state-of-the-art of DQ tools and how DQ measurement is currently perceived in practice by companies in contrast to DQ research.

So far, there are only a few surveys on DQ tools in general, and in particular no survey that investigated the existence of generic DQ metrics. There is also no survey that identified the existence of DQ monitoring capabilities in tools. We attempted to close this gap with our survey and provide the results regarding the available DQ metrics and DQ monitoring capabilities for the tools analyzed.

While we identified the need for more *automation* in data profiling and DQ measurement (with respect to initialization as well as continuous DQ monitoring), at the same time, a clear *declaration and explanation* of the performed calculations and algorithms is essential. In several tools (e.g., AggregateProfiler, InfoZoom), plots were generated or outliers were detected without a clear declaration of the used threshold or distance function. In alignment with the requirement for interpretability of data profiling results, we highlight the need for clear declaration of the parameters used.

In our ongoing and future work, we will introduce a practical DQ methodology that regards at directly measurable aspects of DQ in contrast to abstract dimensions with no common understanding. We also think that it is worth investigating the potential for automated out-of-the-box data profiling along with a clear declaration of the used parameters, which might be modified after the initial run. Part of our ongoing research is to exploit time-series analytics for further investigation of DQ monitoring results in order to predict trends and sudden changes in the DQ (as suggested in Ehrlinger and Wöß, 2017). Since a deep investigation of single DP requirements was out of scope for this survey, it would also be worth to further investigate specific implementations and their proper functionality, for example, which aspects yield floating point differences. Last but not least, further investigation of the 339 excluded domain-specific DQ tools with regard to their domains and their scope would be interesting.

The top vendors of DQ tools worldwide have between 7,200 (Experian), 5,000 (Informatica) and 2,700 (SAS) customers for their DQ product line (Chien and Jain, 2019). Compared to the hype for AI and ML, these low numbers show high catch-up demand for DQ tool applications in general.

## Data Availability Statement

The raw data supporting the conclusions of this article will be made available by the authors, without undue reservation.

## Author Contributions

LE designed and conducted the survey. LE and WW wrote this article. Both authors contributed to the article and approved the submitted version.

## Funding

The research reported in this paper has been supported by the Austrian Ministry for Transport, Innovation and Technology, the Federal Ministry for Digital and Economic Affairs, and the State of Upper Austria in the frame of the COMET center SCCH.

## Conflict of Interest

The authors declare that the research was conducted in the absence of any commercial or financial relationships that could be construed as a potential conflict of interest.

## Publisher's Note

All claims expressed in this article are solely those of the authors and do not necessarily represent those of their affiliated organizations, or those of the publisher, the editors and the reviewers. Any product that may be evaluated in this article, or claim that may be made by its manufacturer, is not guaranteed or endorsed by the publisher.

## Footnotes

^1^https://github.com/CanburakTumer/SQL-Utils (January, 2022).

^2^https://github.com/rogelj/DescribeCol (January, 2022).

^3^http://www.dofactory.com/sql/sample-database (January, 2022).

^4^https://sourceforge.net/projects/dataquality (January, 2022).

^5^https://griffin.apache.org (January, 2022).

^6^https://one.ataccama.com (January, 2022).

^7^http://www.datamartist.com (January, 2022).

^8^https://www.ibm.com/support/knowledgecenter/en/SSZJPZ_11.7.0/com.ibm.swg.im.iis.productization.iisinfsv.install.doc/topics/cont_iisinfsrv_install.html (January, 2022).

^9^http://www.humanit.de (January, 2022).

^10^https://github.com/mobydq/mobydq (January, 2022).

^11^http://openrefine.org (January, 2022).

^12^https://github.com/OpenRefine/OpenRefine (January, 2022).

^13^http://www.oracle.com/technetwork/middleware/oedq/downloads/edq-vm-download-2424092.html (January, 2022).

^14^https://github.com/Talend/tdq-studio-se (January, 2022).

^15^http://www.sas.com (January, 2022).

^16^https://info.talend.com/vrstosdq_150602.html (January, 2022).

^17^https://community.talend.com (January, 2022).

^18^http://web.mit.edu/tdqm (January, 2022).
